# Supplementary figures and images for: Eco1-dependent cohesin acetylation anchors chromatin loops and cohesion to define functional meiotic chromosome domains
Source: eLife. 2022 Feb 1;11:e74447. doi: 10.7554/eLife.74447 (PMC8856730; doi:10.7554/eLife.74447)

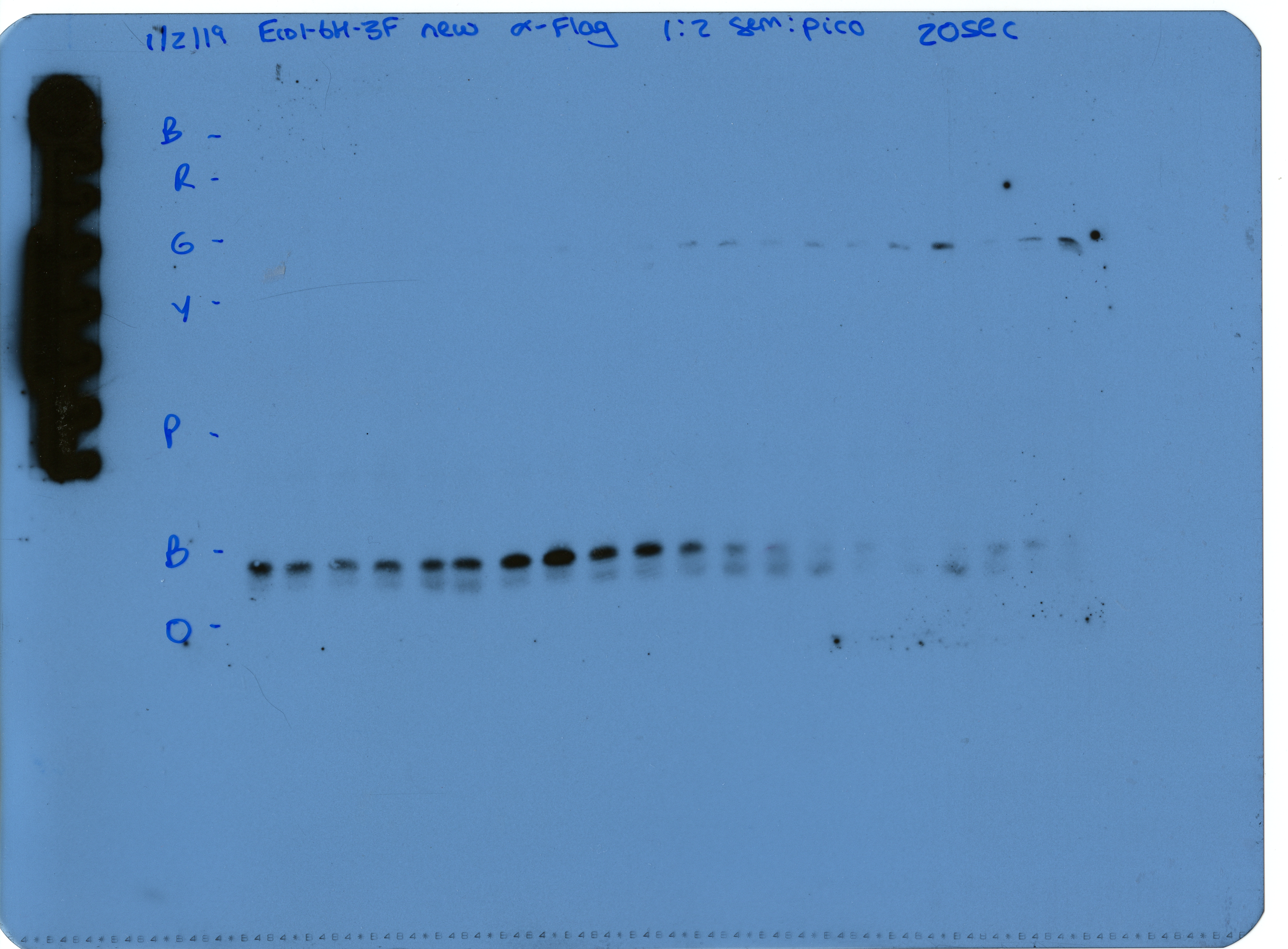

Supplement: Figure 1—source data 1. [file elife-74447-fig1-data1.zip › Figure 1-source data 1/Figure 1-source data 1.tif]

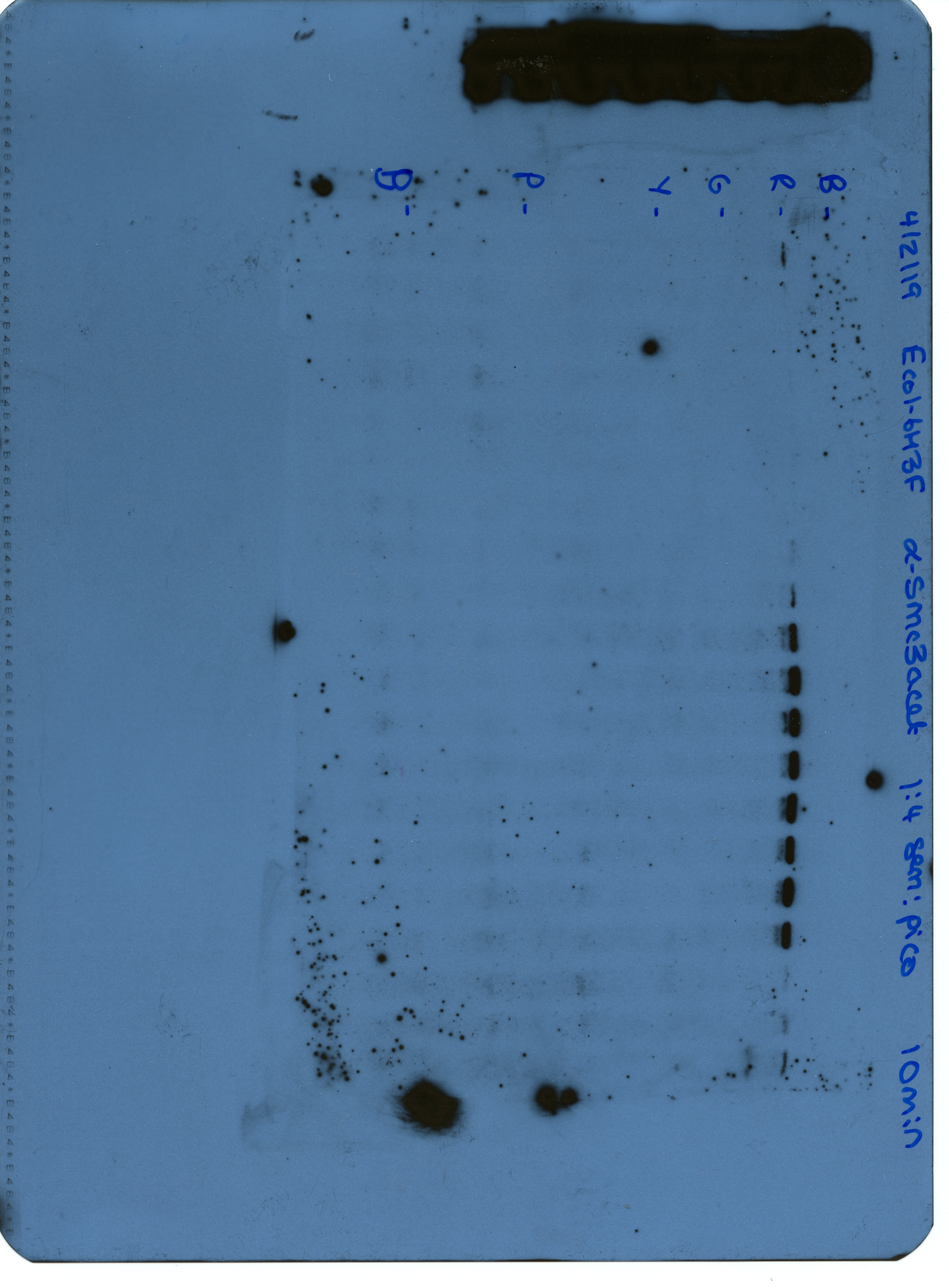

Supplement: Figure 1—source data 2. [file elife-74447-fig1-data2.zip › Figure 1-source data 2/Figure 1-source data 2.tif]

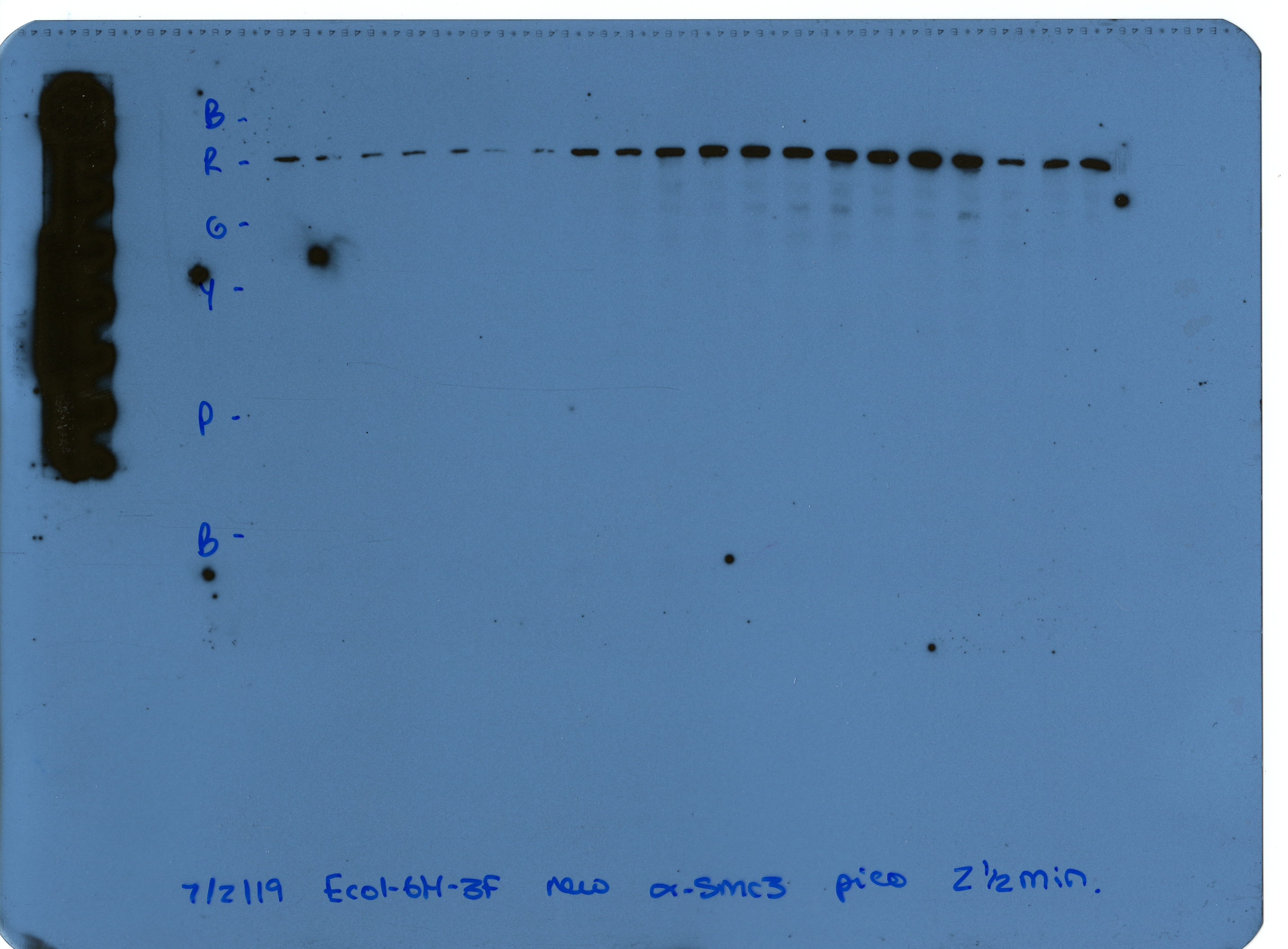

Supplement: Figure 1—source data 3. [file elife-74447-fig1-data3.zip › Figure 1-source data 3/Figure 1-source data 3.tif]

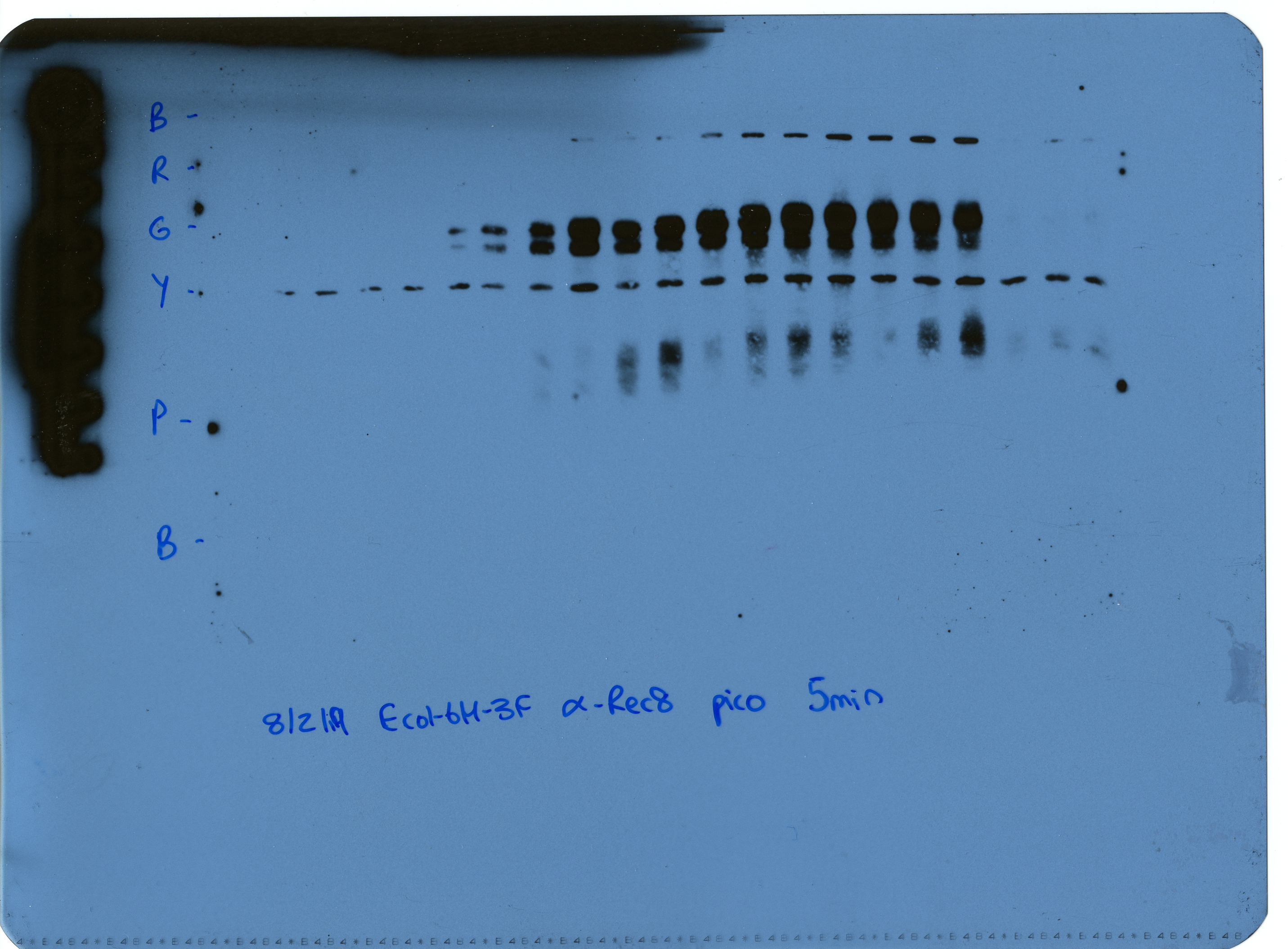

Supplement: Figure 1—source data 4. [file elife-74447-fig1-data4.zip › Figure 1-source data 4/Figure 1-source data 4.tif]

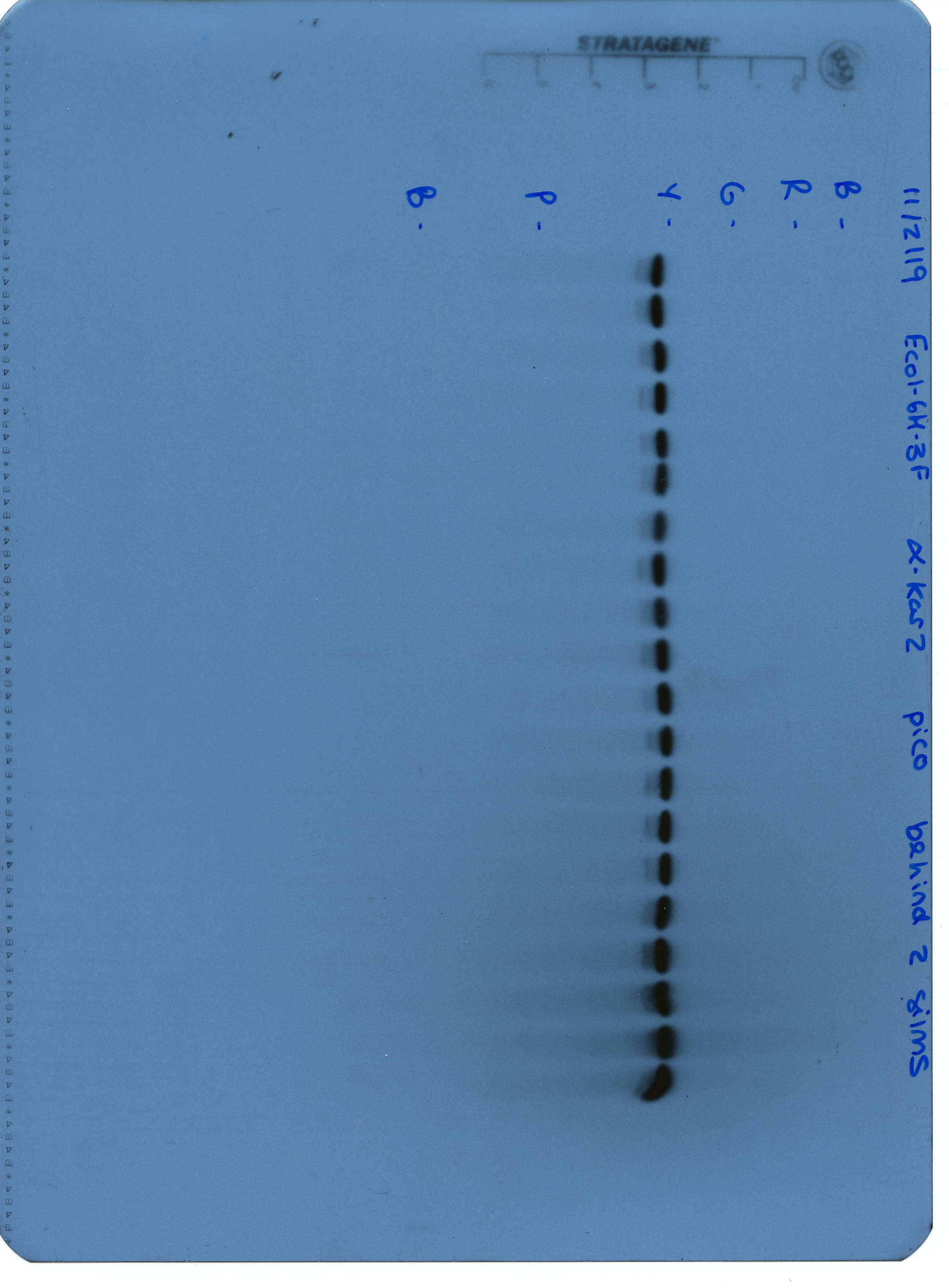

Supplement: Figure 1—source data 5. [file elife-74447-fig1-data5.zip › Figure 1-source data 5/Figure 1-source data 5.tif]

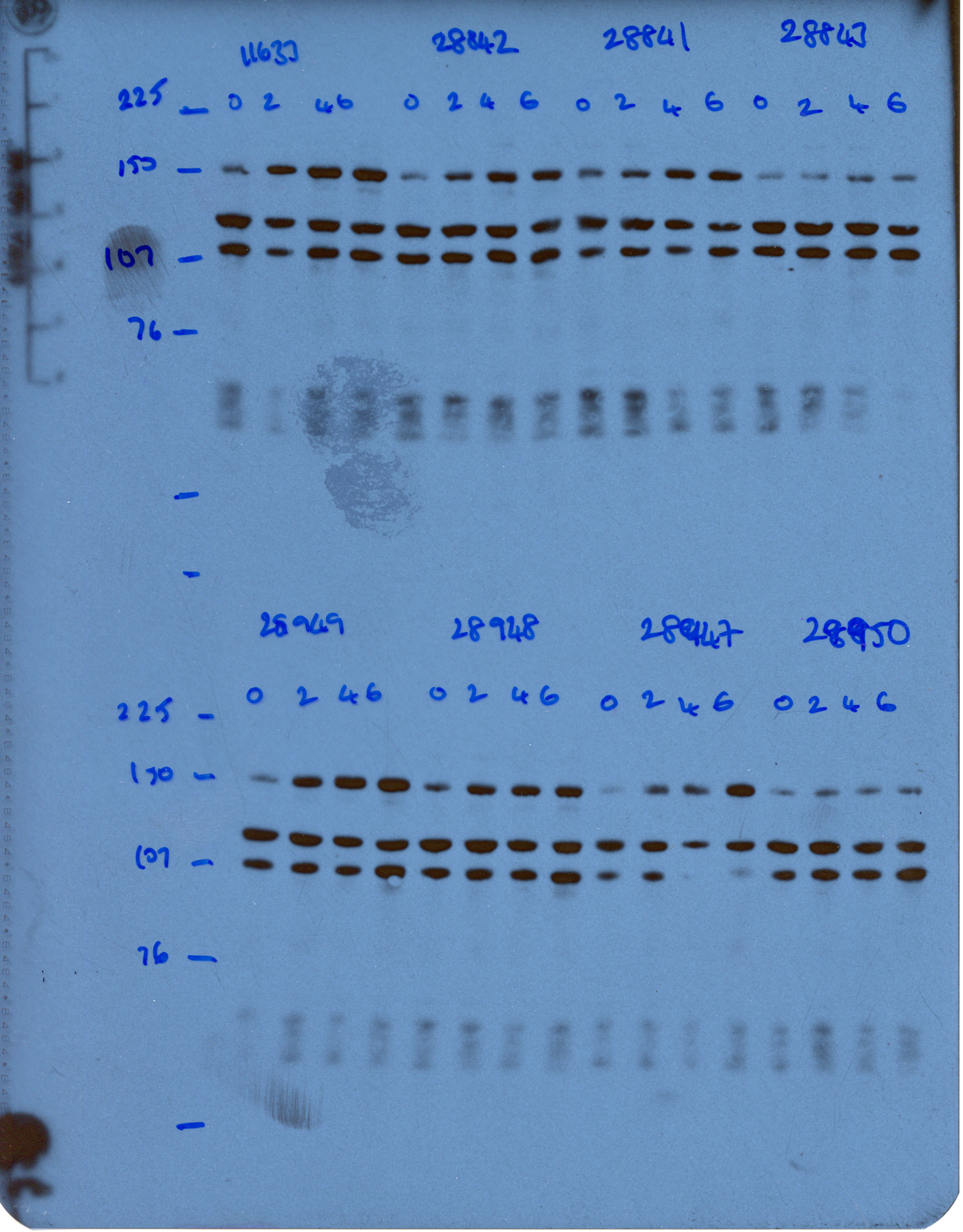

Supplement: Figure 1—source data 6. [file elife-74447-fig1-data6.zip › Figure 1-source data 6/Figure 1-source data 6.tif]

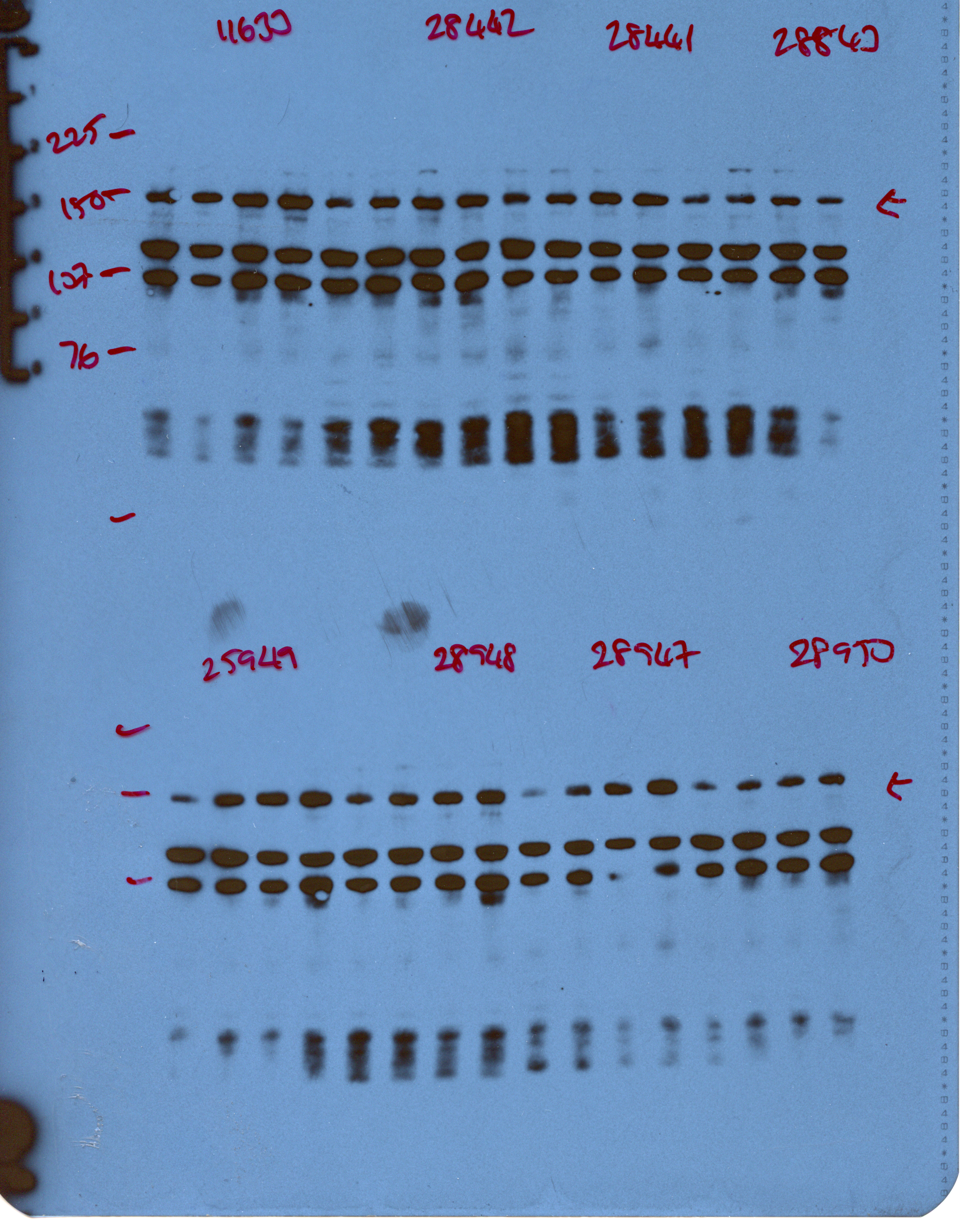

Supplement: Figure 1—source data 7. [file elife-74447-fig1-data7.zip › Figure 1-source data 7/Figure 1-source data 7.tif]

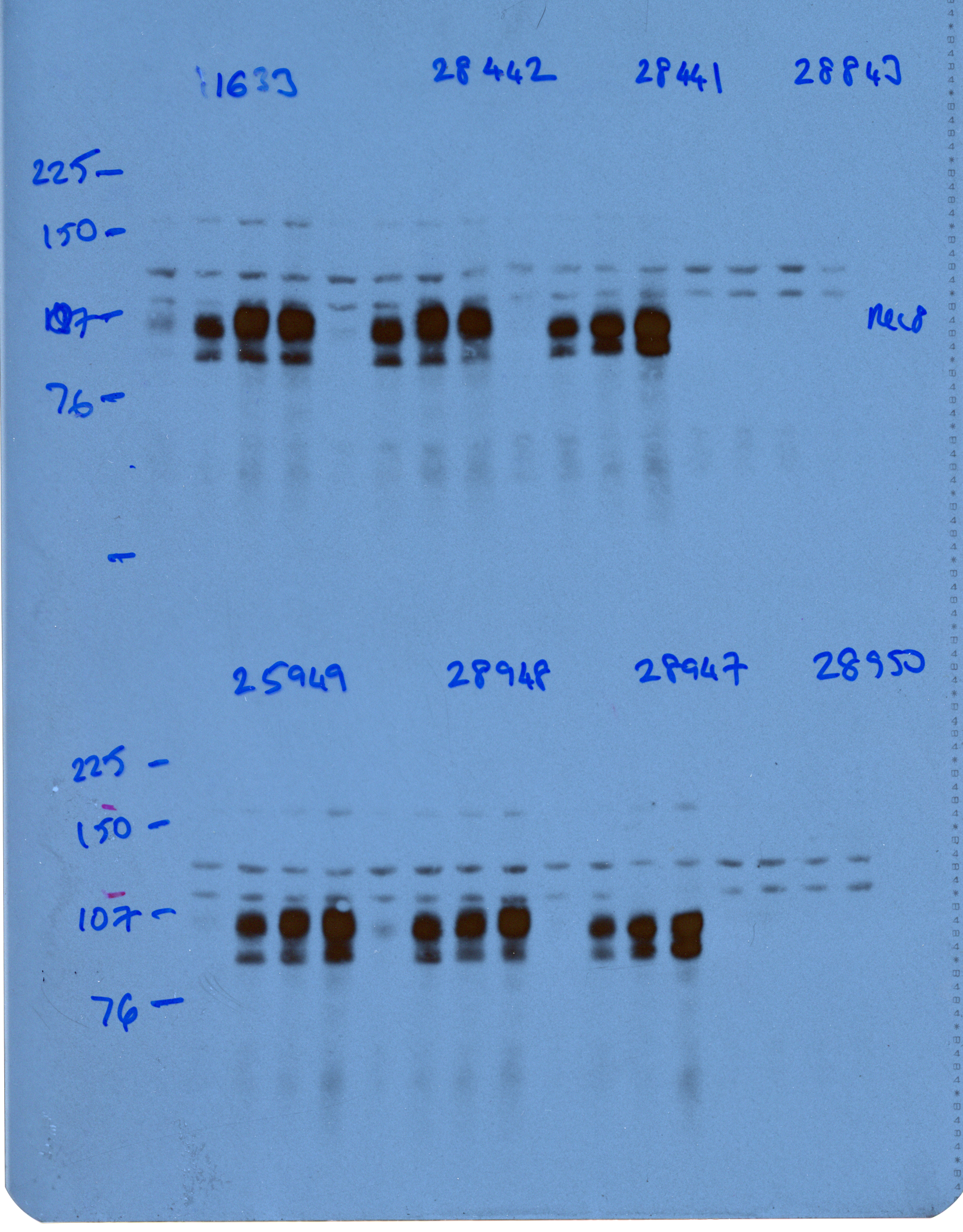

Supplement: Figure 1—source data 8. [file elife-74447-fig1-data8.zip › Figure 1-source data 8/Figure 1-source data 8.tif]

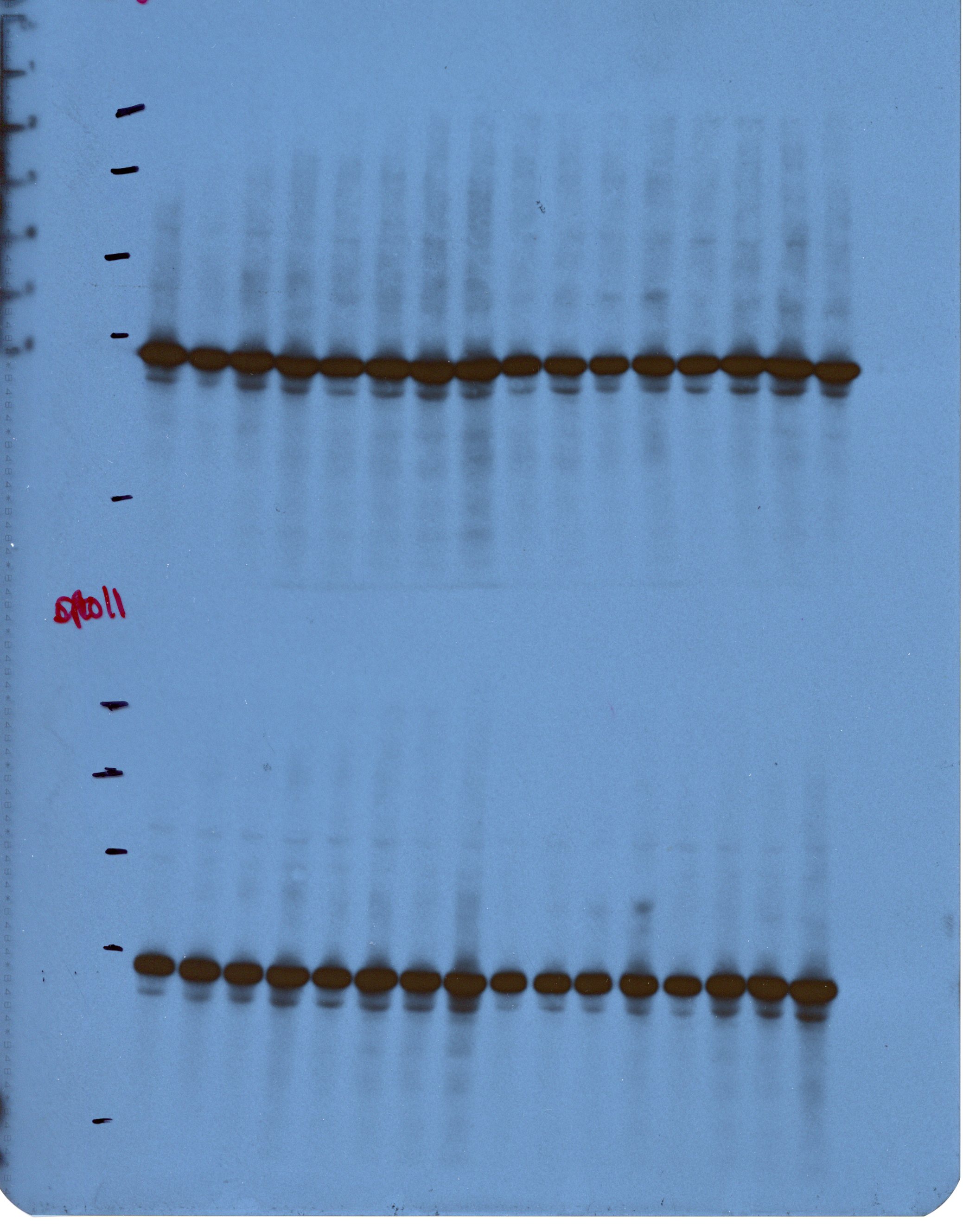

Supplement: Figure 1—source data 9. [file elife-74447-fig1-data9.zip › Figure 1-source data 9/Figure 1-source data 9.tif]

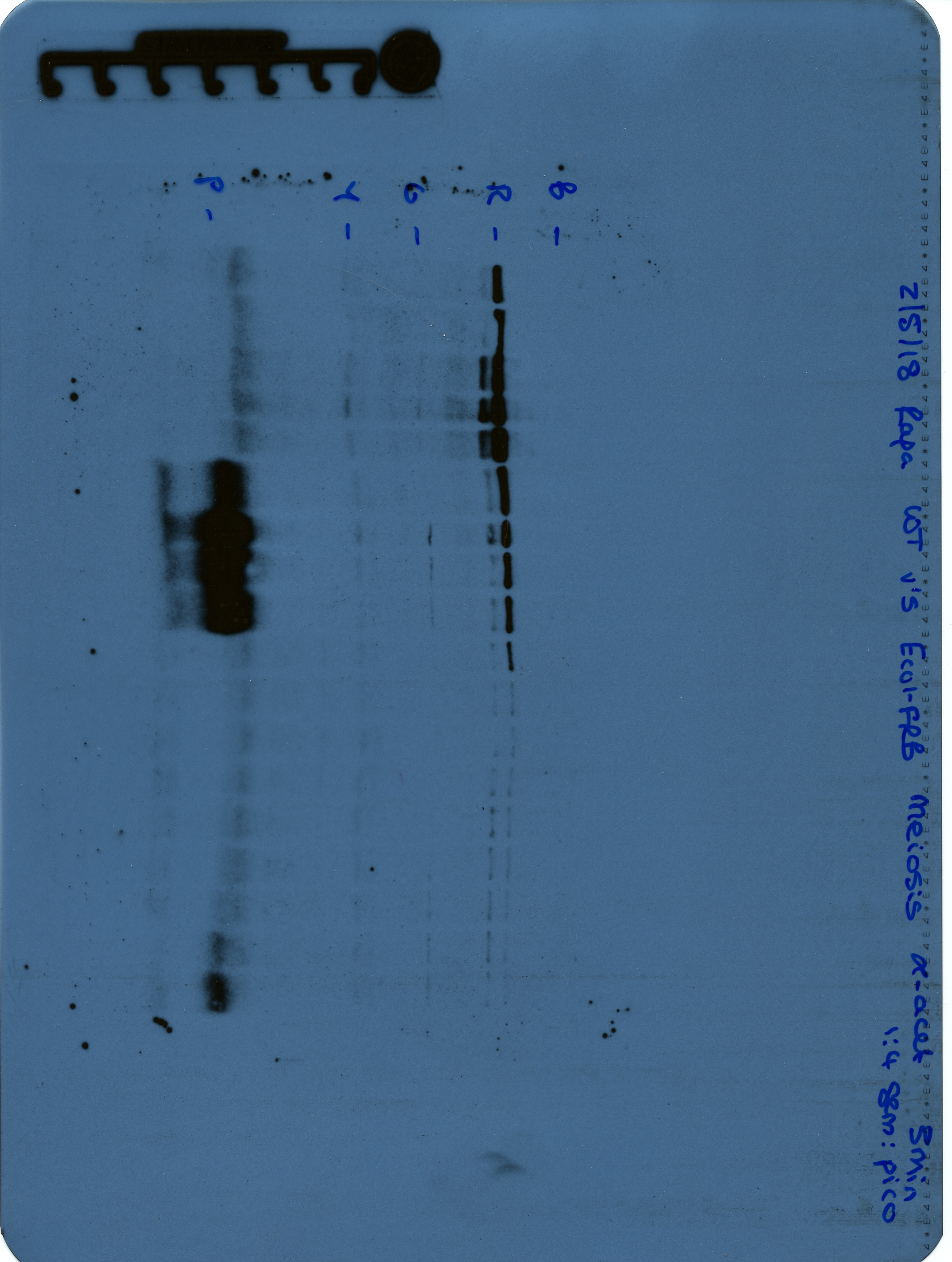

Supplement: Figure 1—source data 10. [file elife-74447-fig1-data10.zip › Figure 1-source data 10/Figure 1-source data 10.tif]

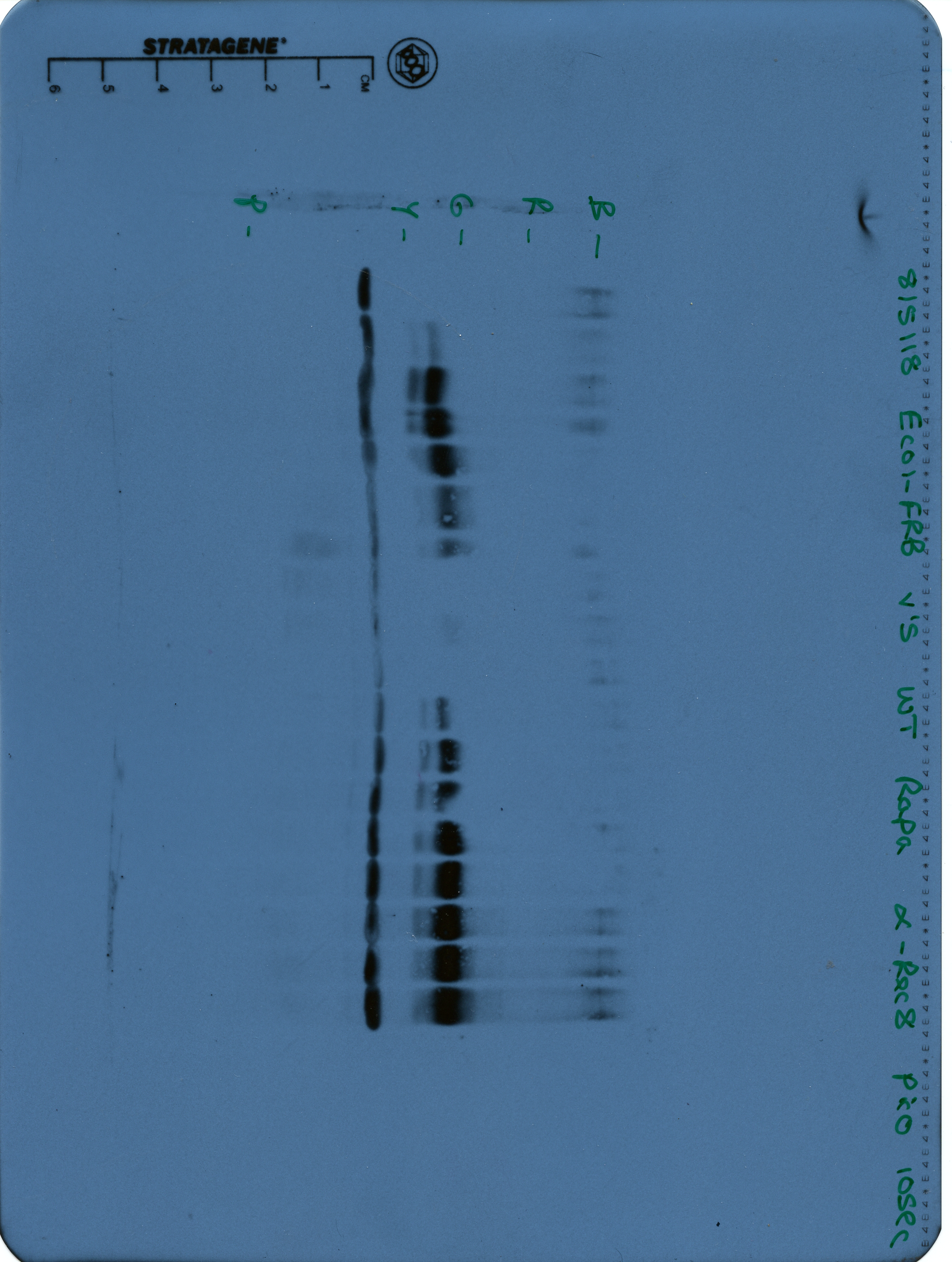

Supplement: Figure 1—source data 11. [file elife-74447-fig1-data11.zip › Figure 1-source data 11/Figure 1-source data 11.tif]

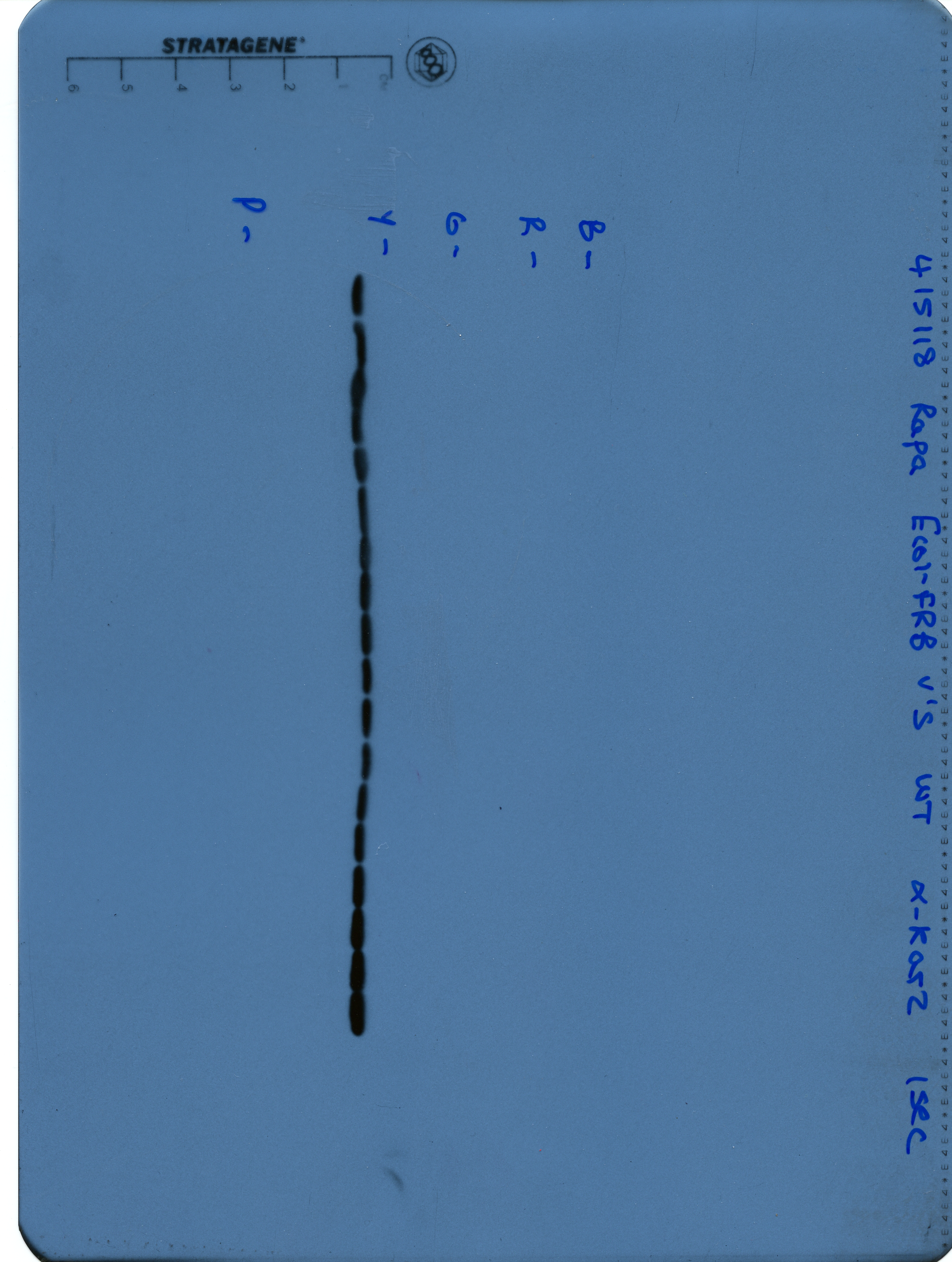

Supplement: Figure 1—source data 12. [file elife-74447-fig1-data12.zip › Figure 1-source data 12/Figure 1-source data 12.tif]

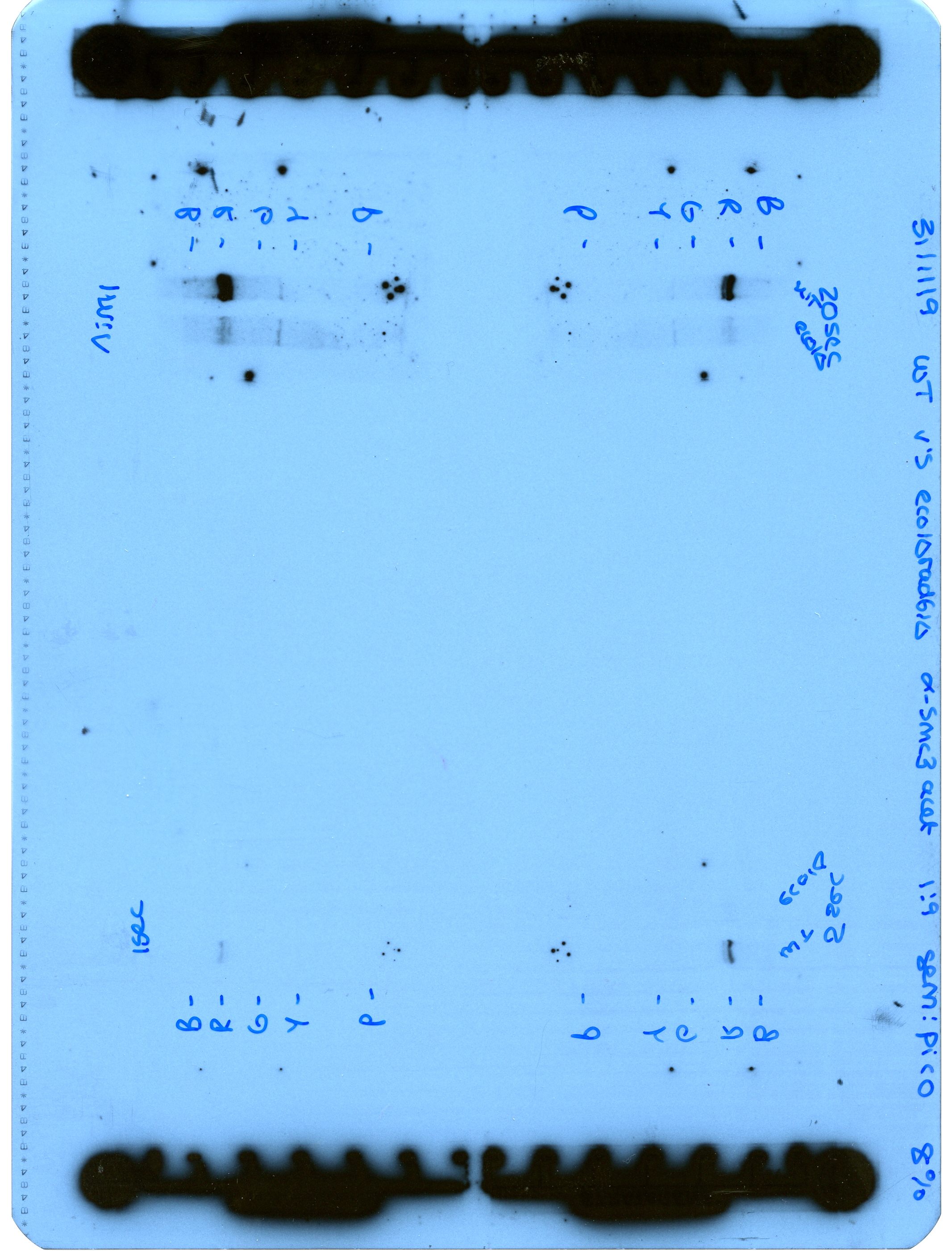

Supplement: Figure 1—figure supplement 1—source data 1. [file elife-74447-fig1-figsupp1-data1.zip › Figure 1-figure supplement 1-source data 1/Figure 1-figure supplement 1-source data 1.tif]

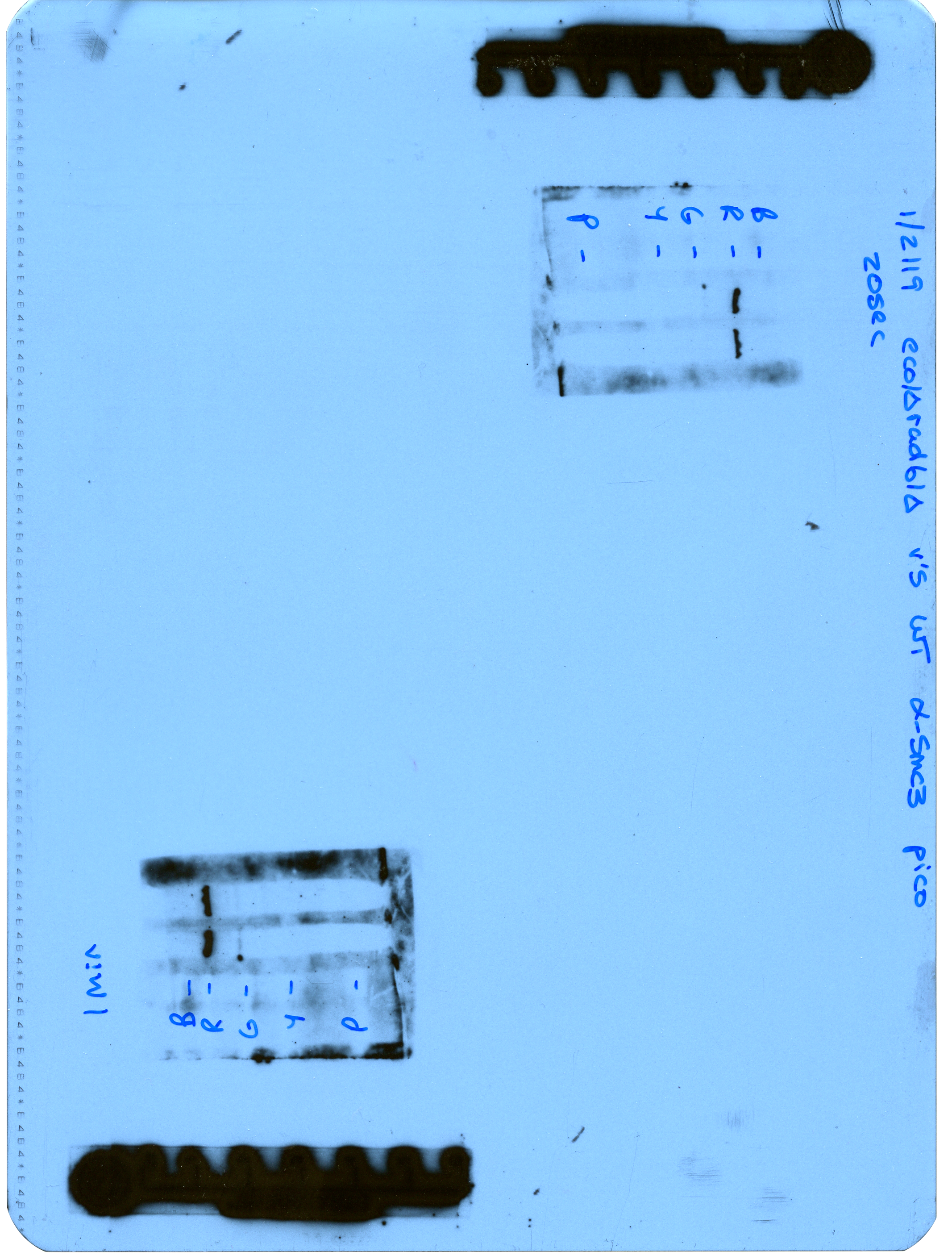

Supplement: Figure 1—figure supplement 1—source data 2. [file elife-74447-fig1-figsupp1-data2.zip › Figure 1-figure supplement 1-source data 2/Figure 1-figure supplement 1-source data 2.tif]

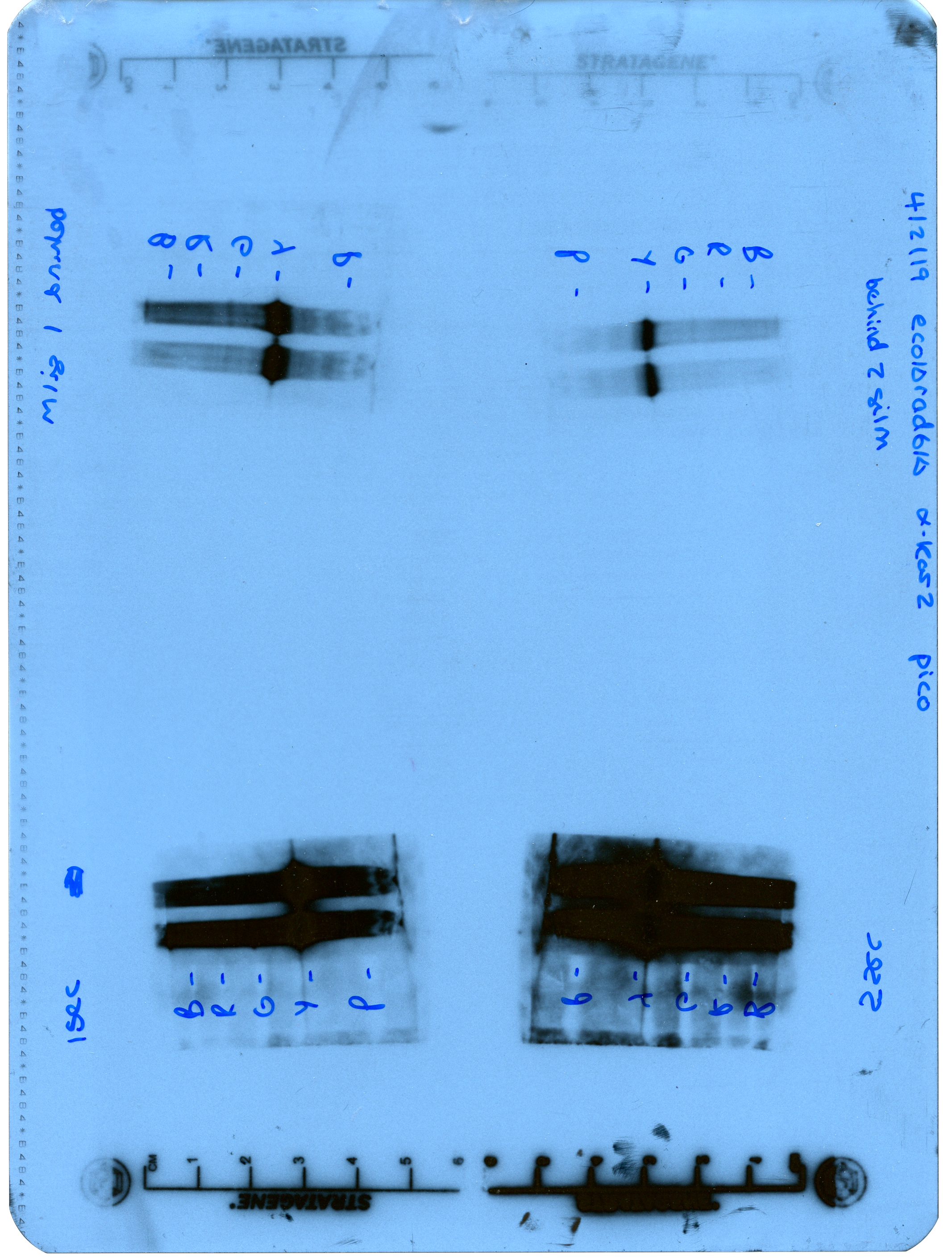

Supplement: Figure 1—figure supplement 1—source data 3. [file elife-74447-fig1-figsupp1-data3.zip › Figure 1-figure supplement 1-source data 3/Figure 1-figure supplement 1-source data 3.tif]

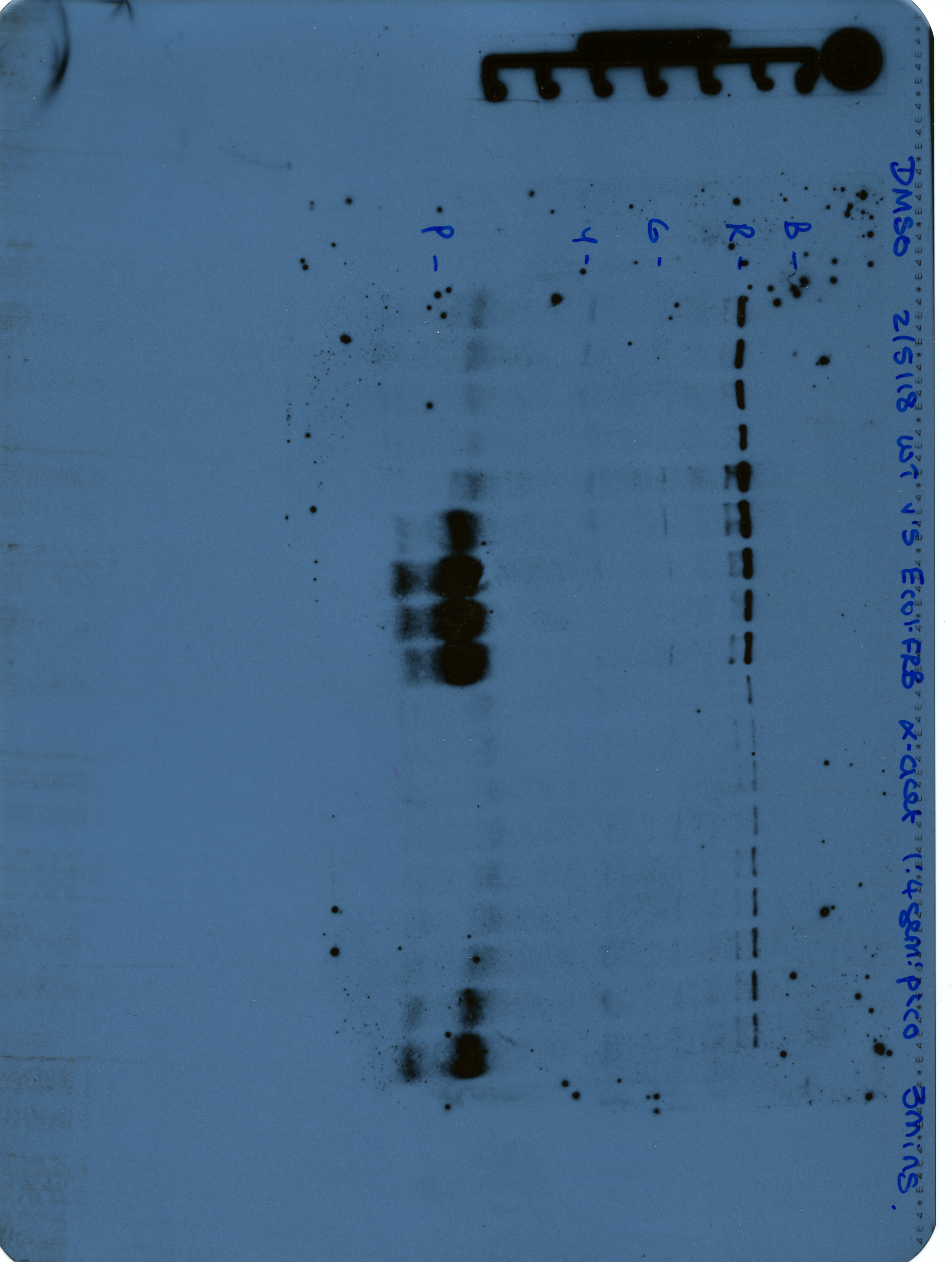

Supplement: Figure 1—figure supplement 3—source data 1. [file elife-74447-fig1-figsupp3-data1.zip › Figure 1-figure supplement 3-source data 1/Figure 1-figure supplement 3-source data 1.tif]

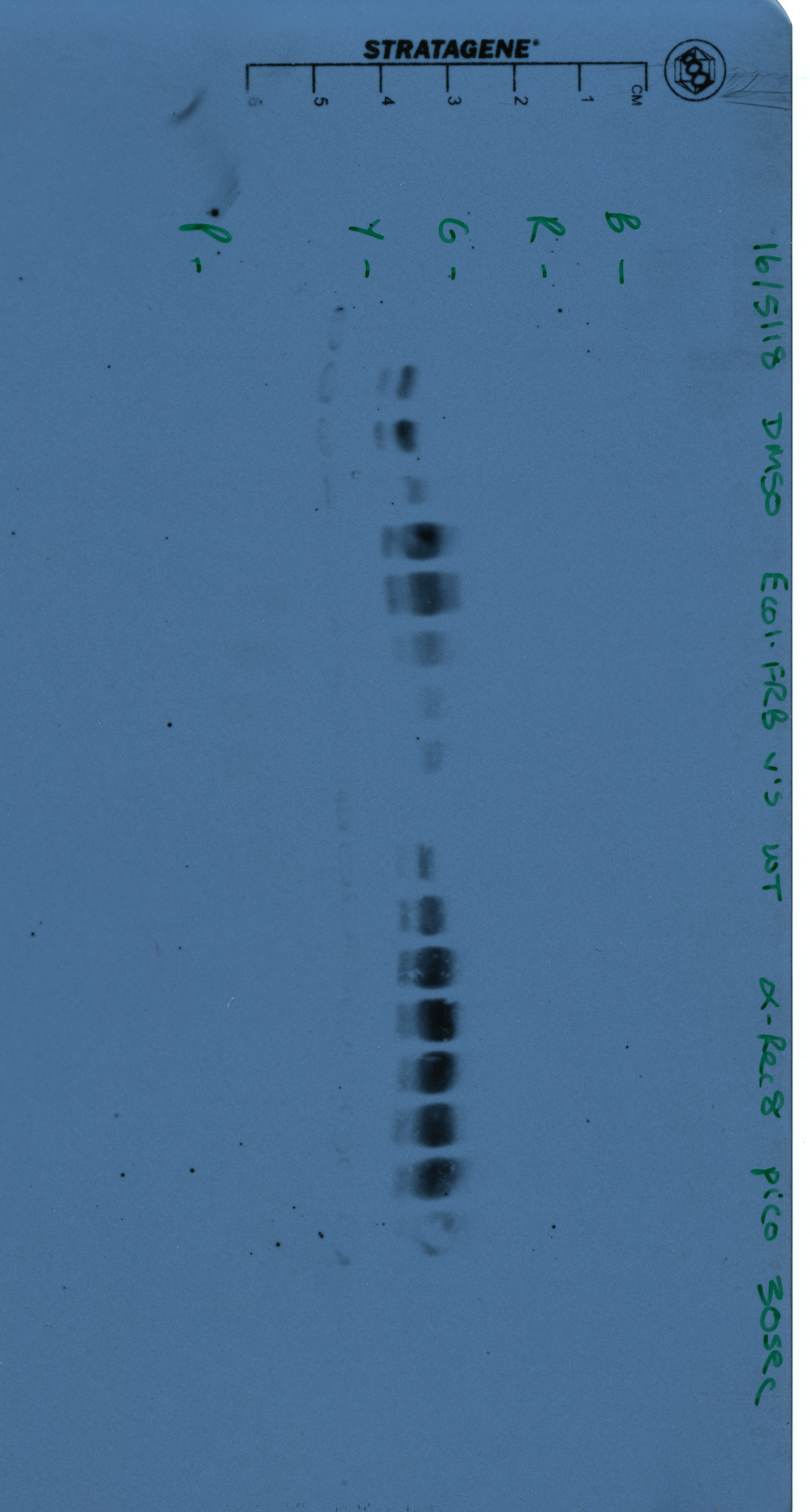

Supplement: Figure 1—figure supplement 3—source data 2. [file elife-74447-fig1-figsupp3-data2.zip › Figure 1-figure supplement 3-source data 2/Figure 1-figure supplement 3-source data 2.tif]

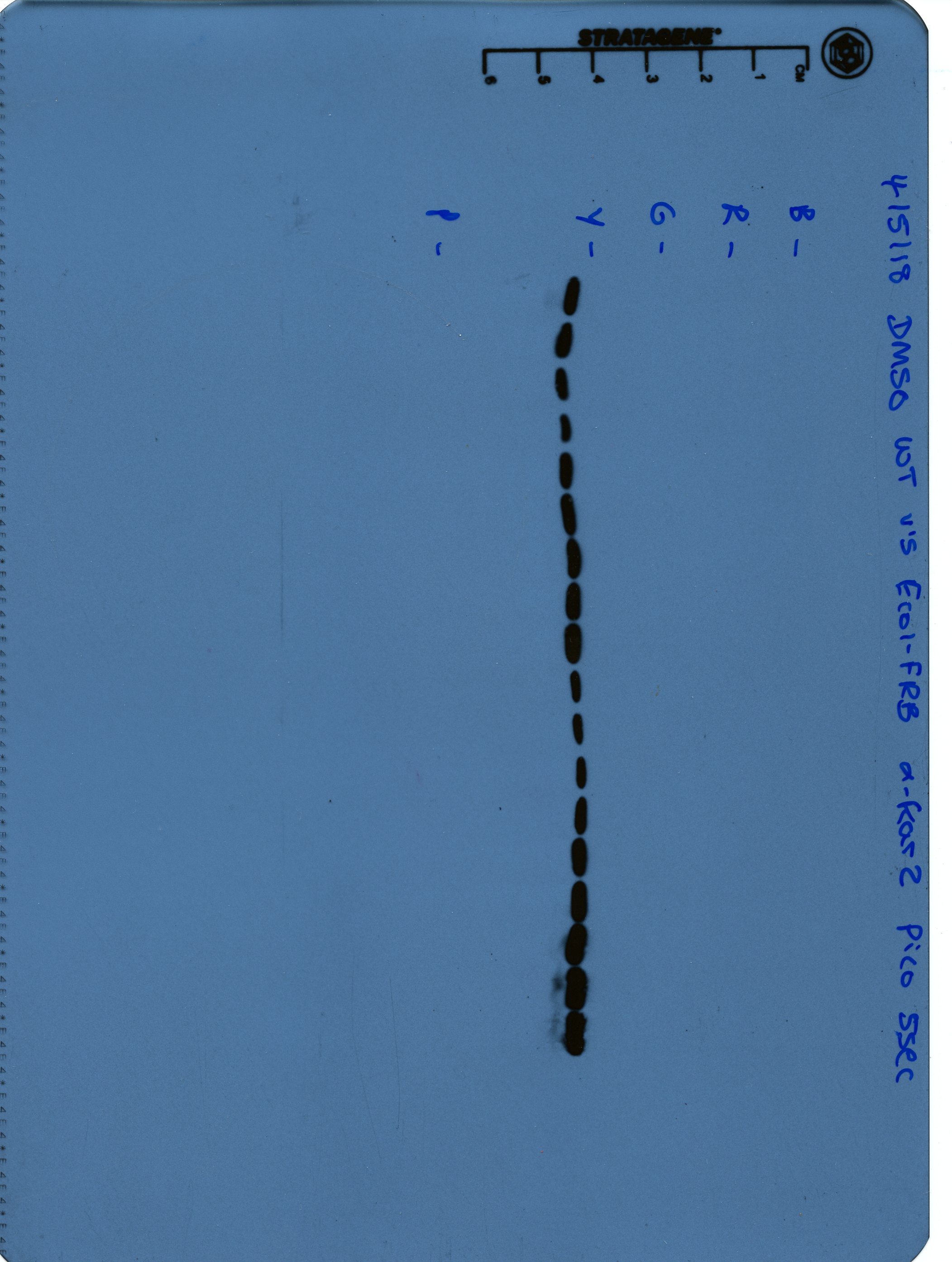

Supplement: Figure 1—figure supplement 3—source data 3. [file elife-74447-fig1-figsupp3-data3.zip › Figure 1-figure supplement 3-source data 3/Figure 1-figure supplement 3-source data 3.tif]

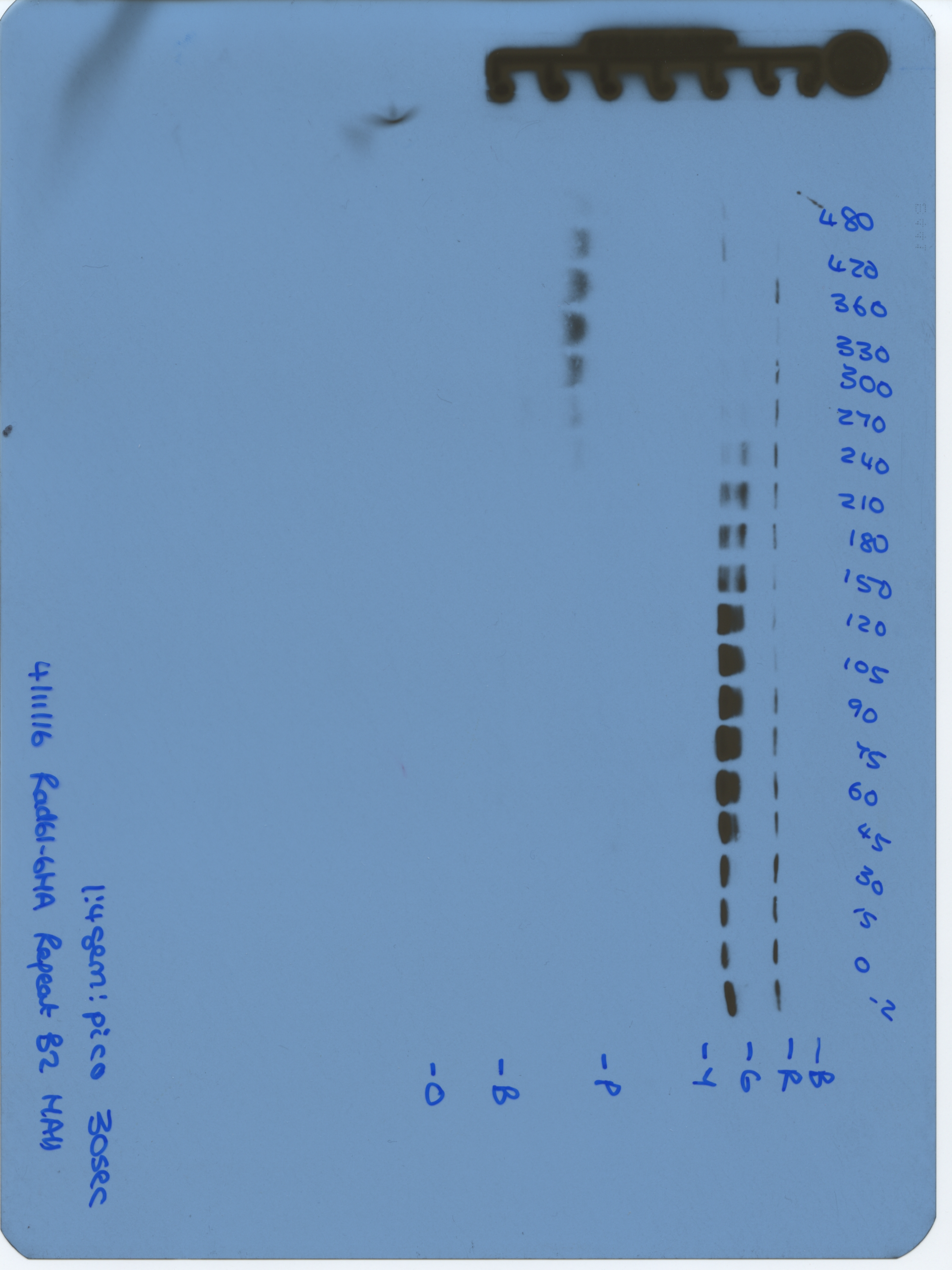

Supplement: Figure 2—source data 1. [file elife-74447-fig2-data1.zip › Figure 2-source data 1/Figure 2-source data 1.tiff]

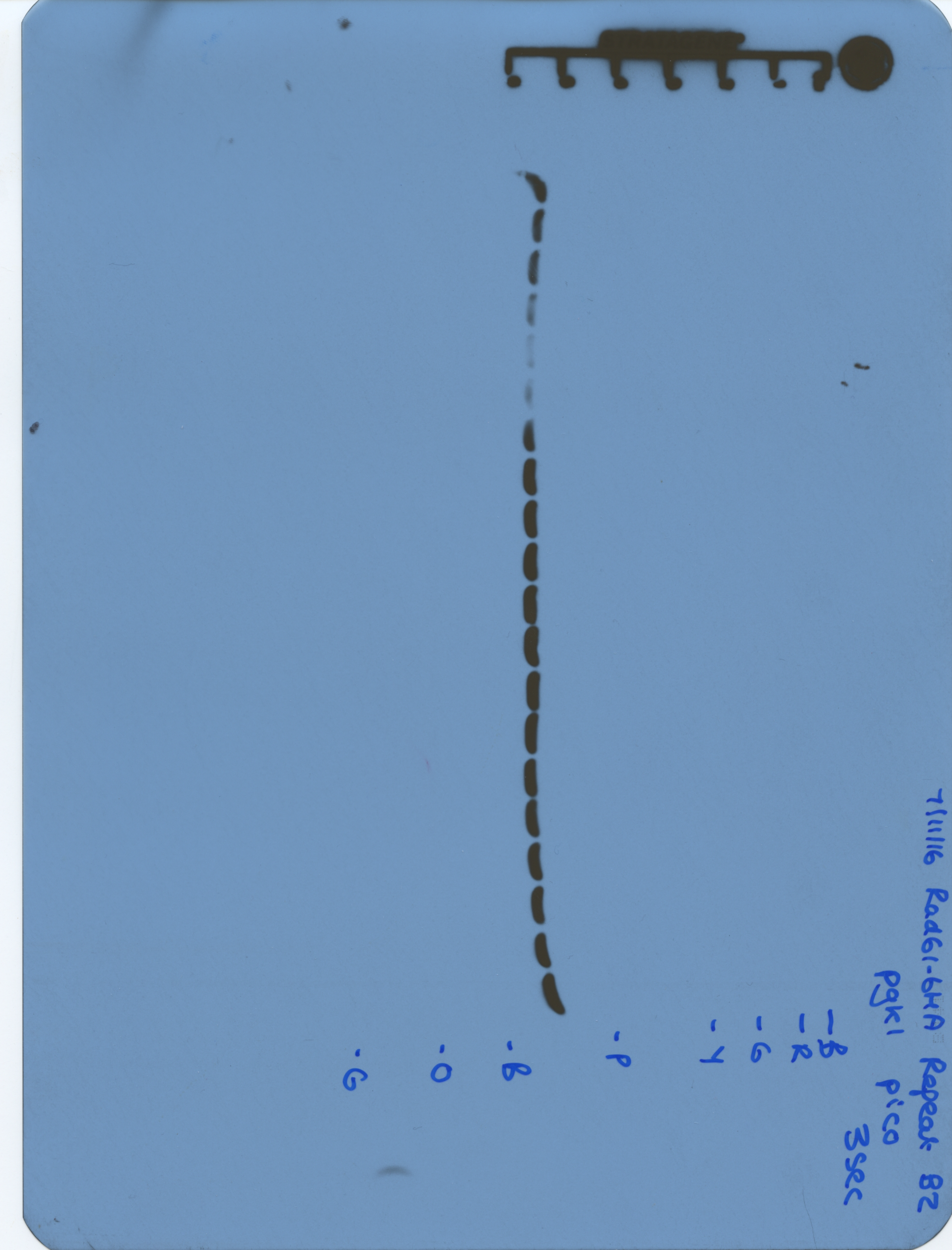

Supplement: Figure 2—source data 2. [file elife-74447-fig2-data2.zip › Figure 2-source data 2/Figure 2-source data 2.tiff]

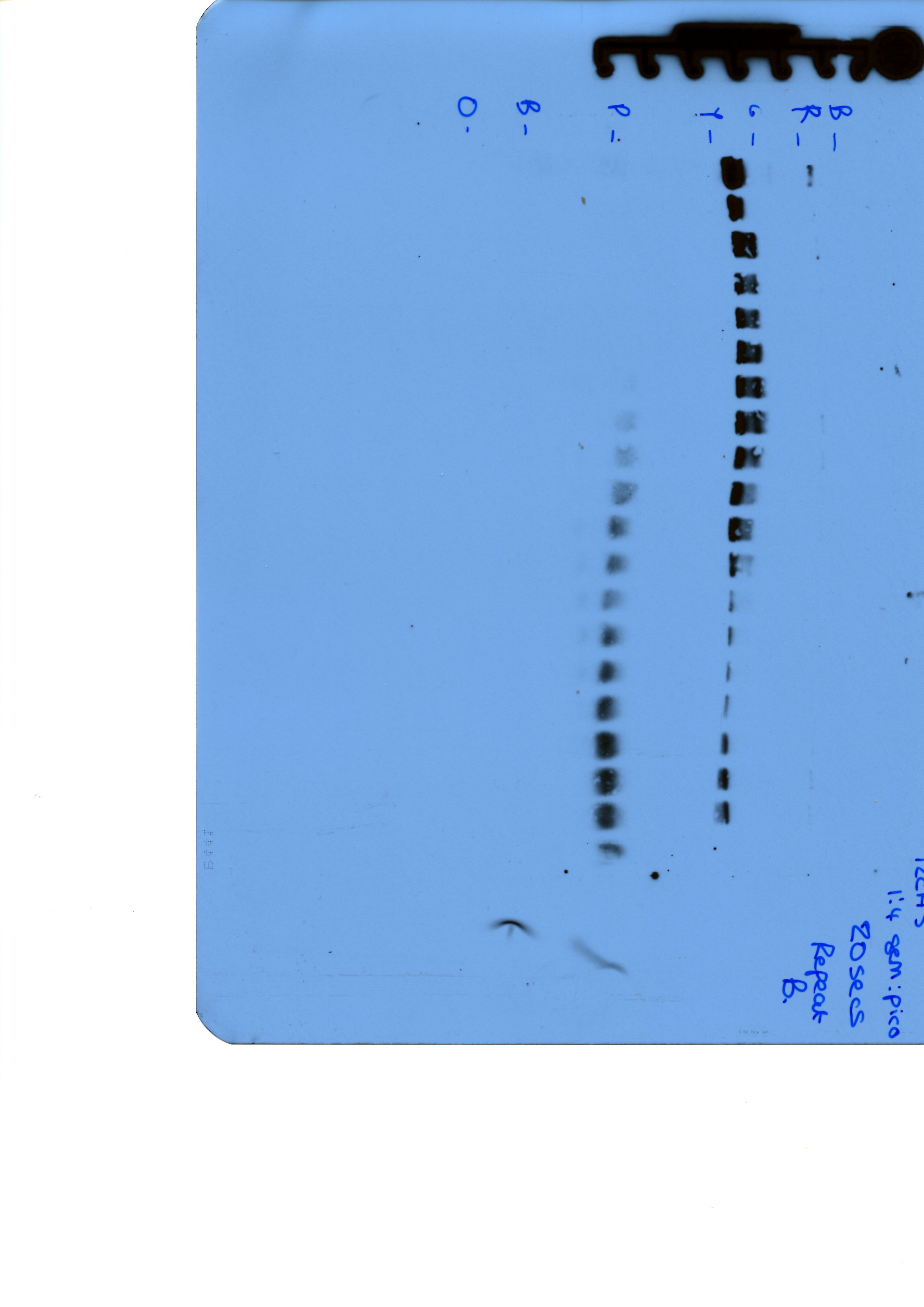

Supplement: Figure 2—figure supplement 1—source data 1. [file elife-74447-fig2-figsupp1-data1.zip › Figure 2-figure supplement 1-source data1/Figure 2-figure supplement 1-source data 1.jpg]

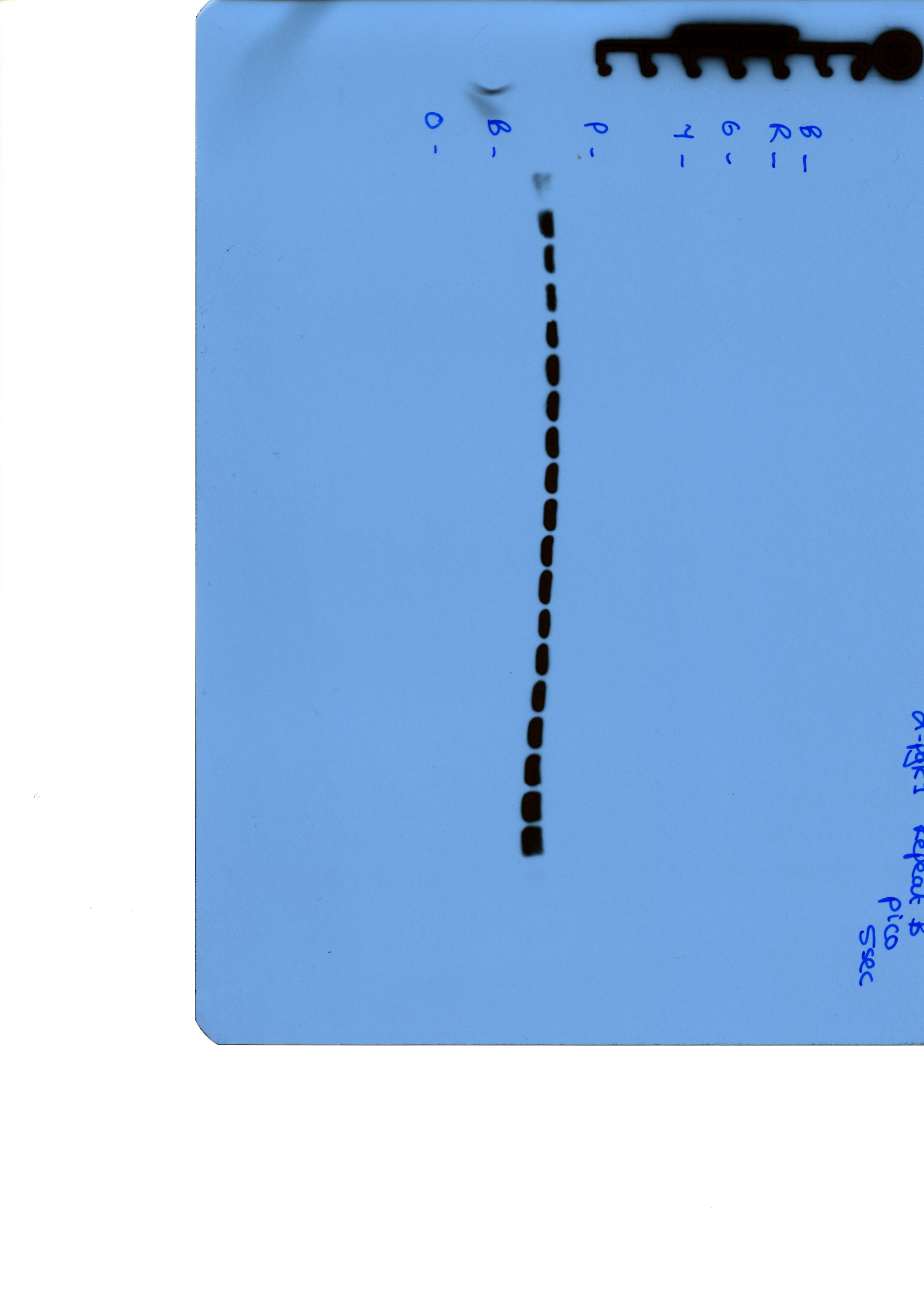

Supplement: Figure 2—figure supplement 1—source data 2. [file elife-74447-fig2-figsupp1-data2.zip › Figure 2-figure supplement 1-source data2/Figure 2-figure supplement 1-source data 2.jpg]

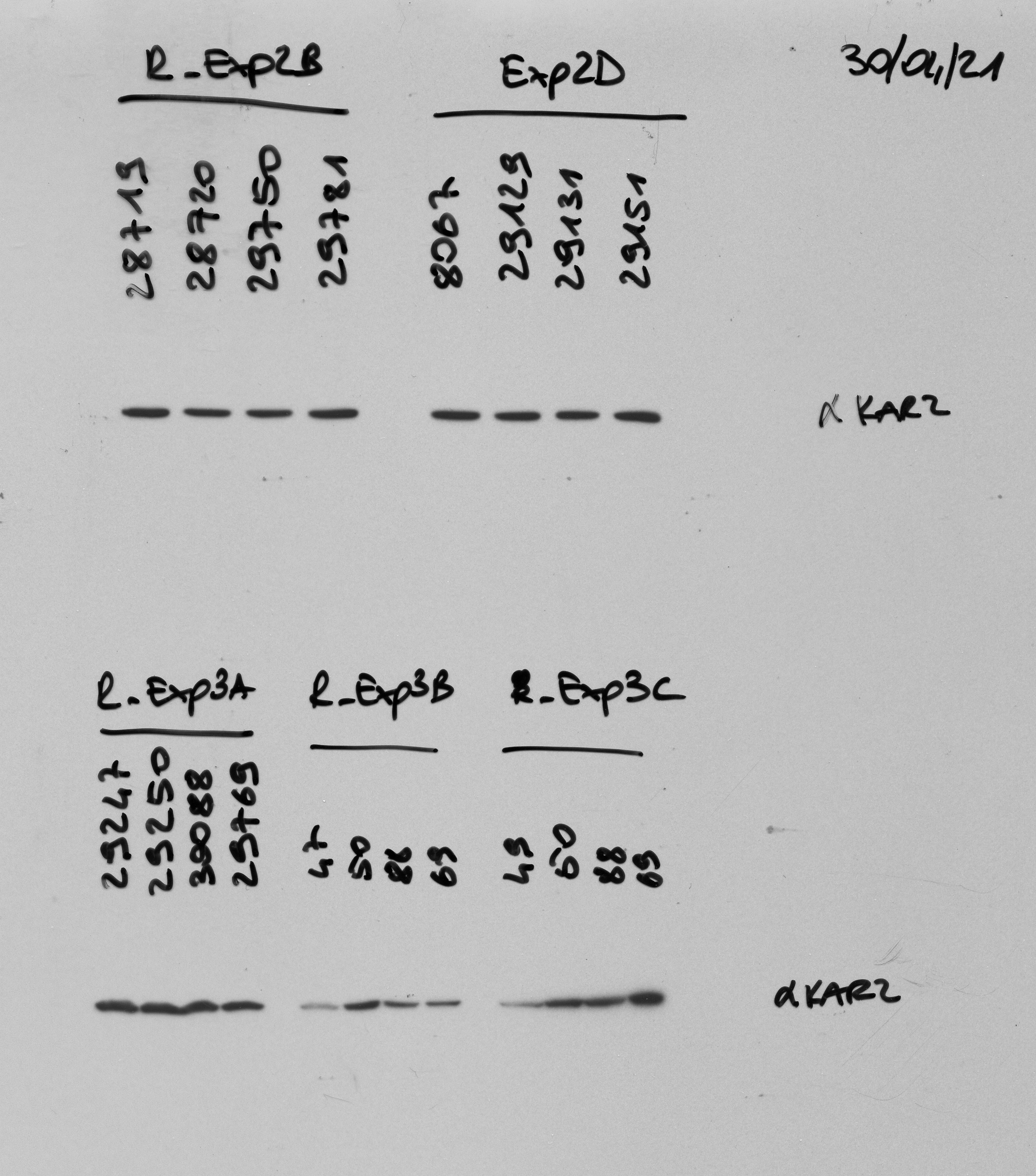

Supplement: Figure 3—source data 1. [file elife-74447-fig3-data1.zip › Figure 3-source data 1/Figure 3-source data 1.tif]

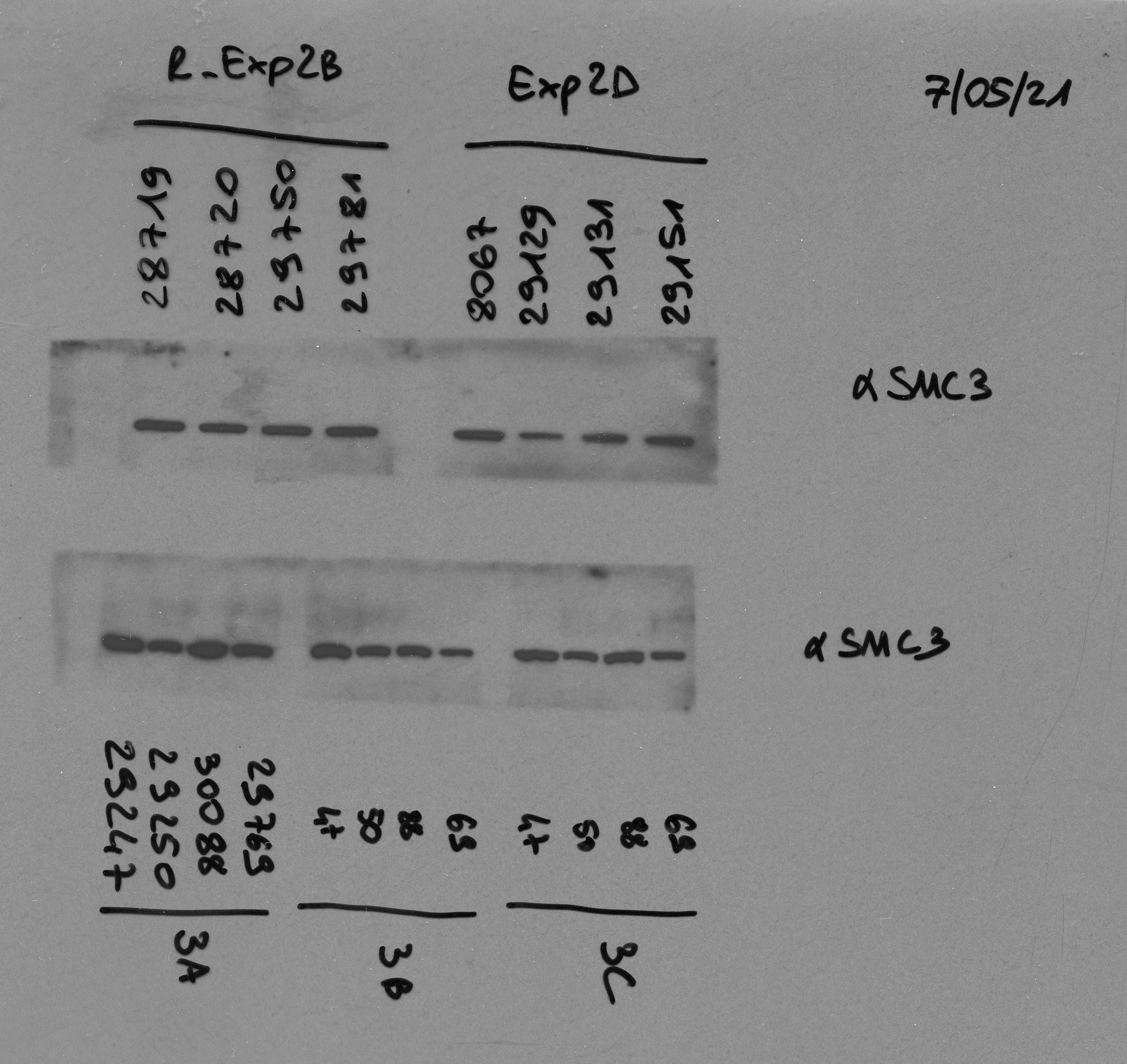

Supplement: Figure 3—source data 2. [file elife-74447-fig3-data2.zip › Figure 3-source data 2/Figure 3-source data 2.tif]

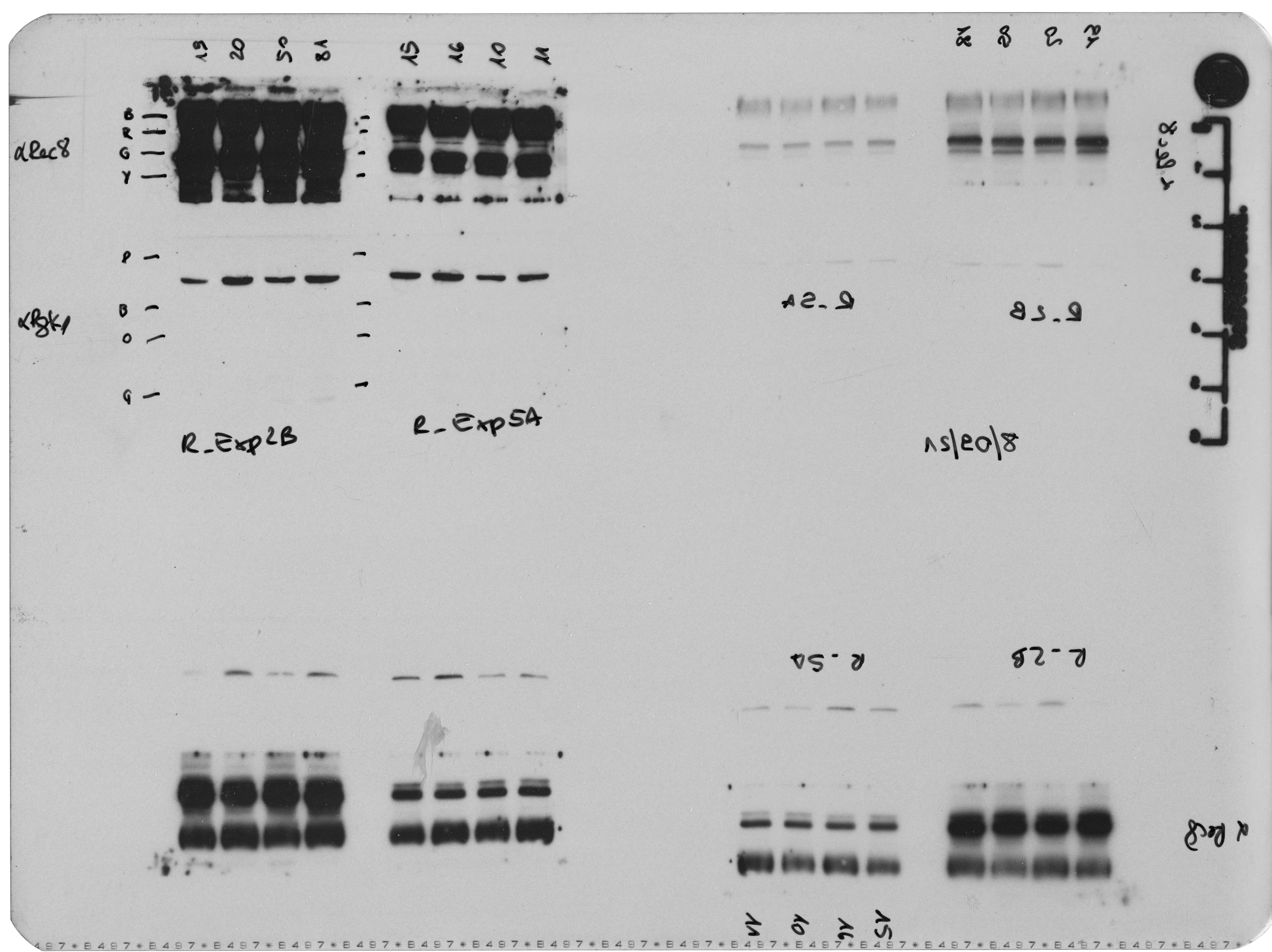

Supplement: Figure 3—source data 3. [file elife-74447-fig3-data3.zip › Figure 3-source data 3/Figure 3-source data 3.tif]

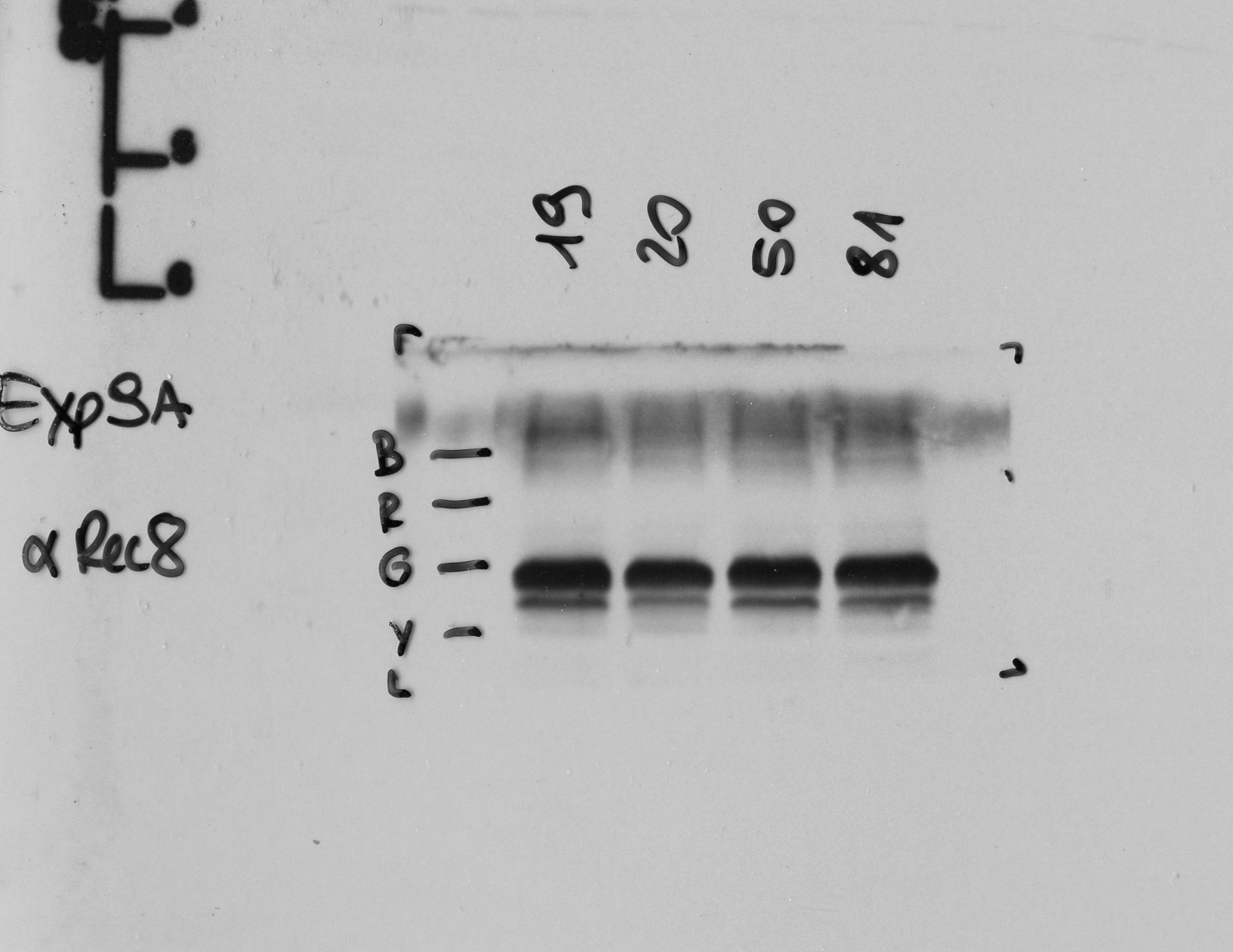

Supplement: Figure 3—figure supplement 1—source data 1. [file elife-74447-fig3-figsupp1-data1.zip › Figure 3-figure supplement 1-source data 1/Figure 3-figure supplement 1-source data 1.tif]

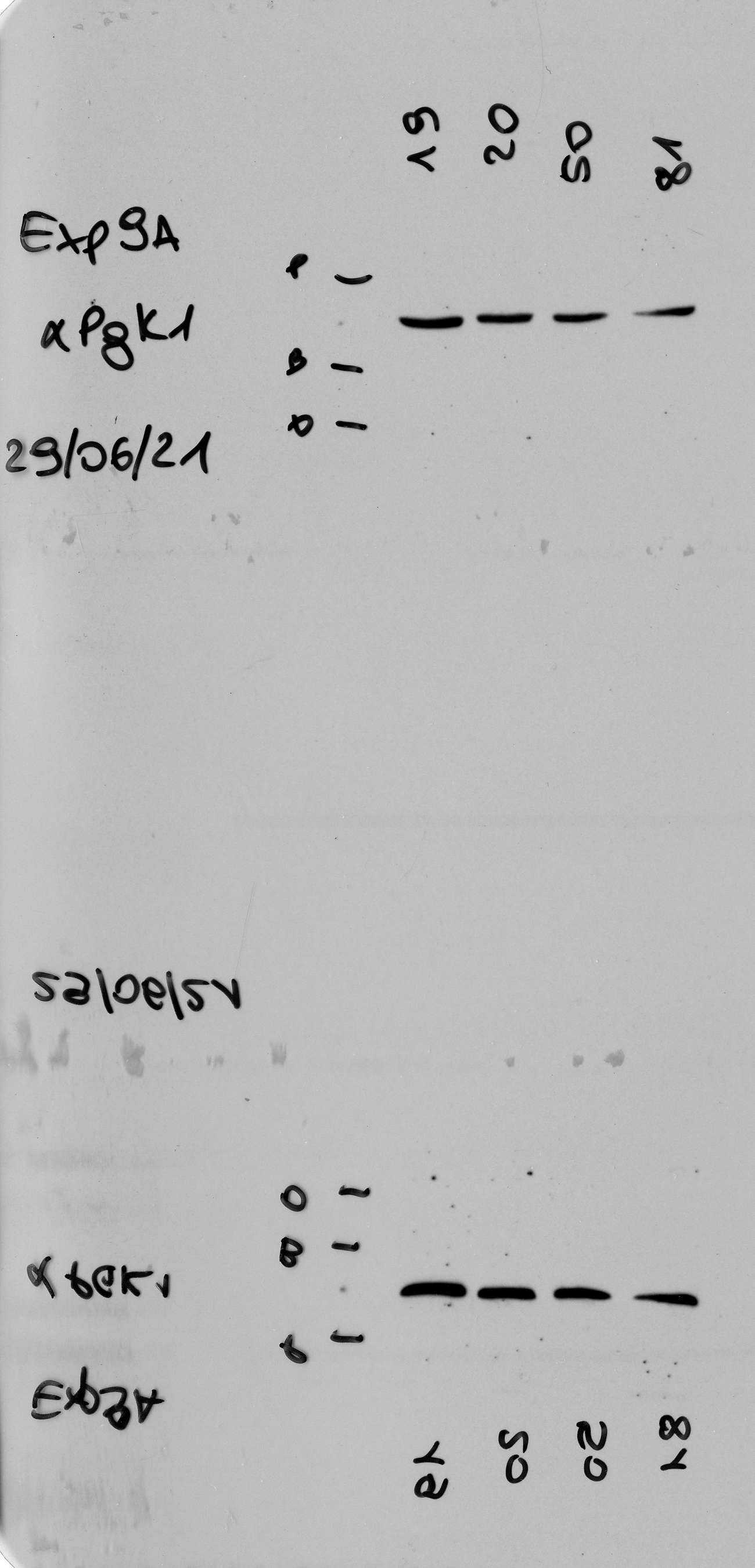

Supplement: Figure 3—figure supplement 1—source data 2. [file elife-74447-fig3-figsupp1-data2.zip › Figure 3-figure supplement 1-source data 2/Figure 3-figure supplement 1-source data 2.tif]

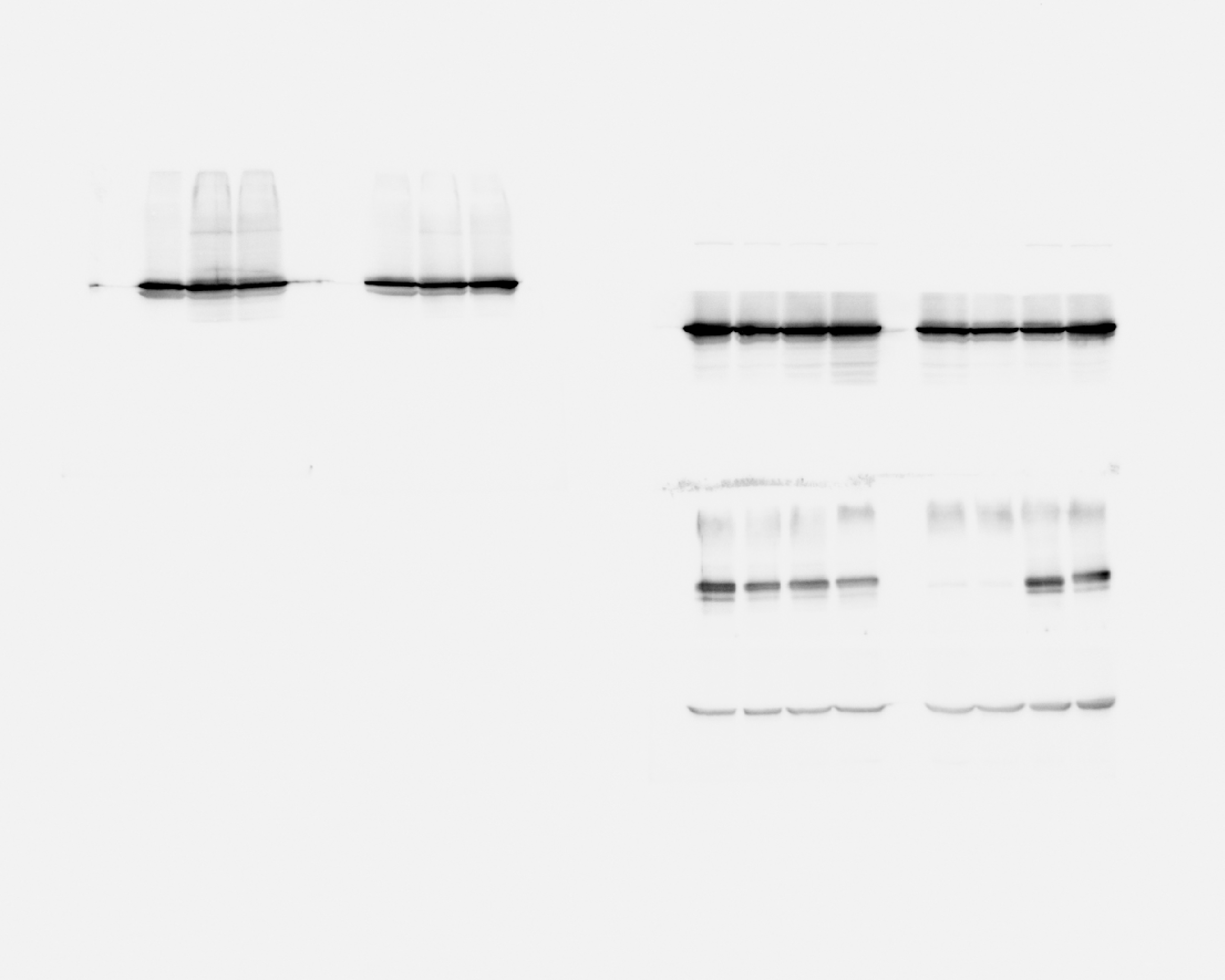

Supplement: Figure 7—figure supplement 1—source data 1. [file elife-74447-fig7-figsupp1-data1.zip › Figure 7-figure supplement 1-source data 1/Figure 7-figure supplement 1-source data 1.tif]

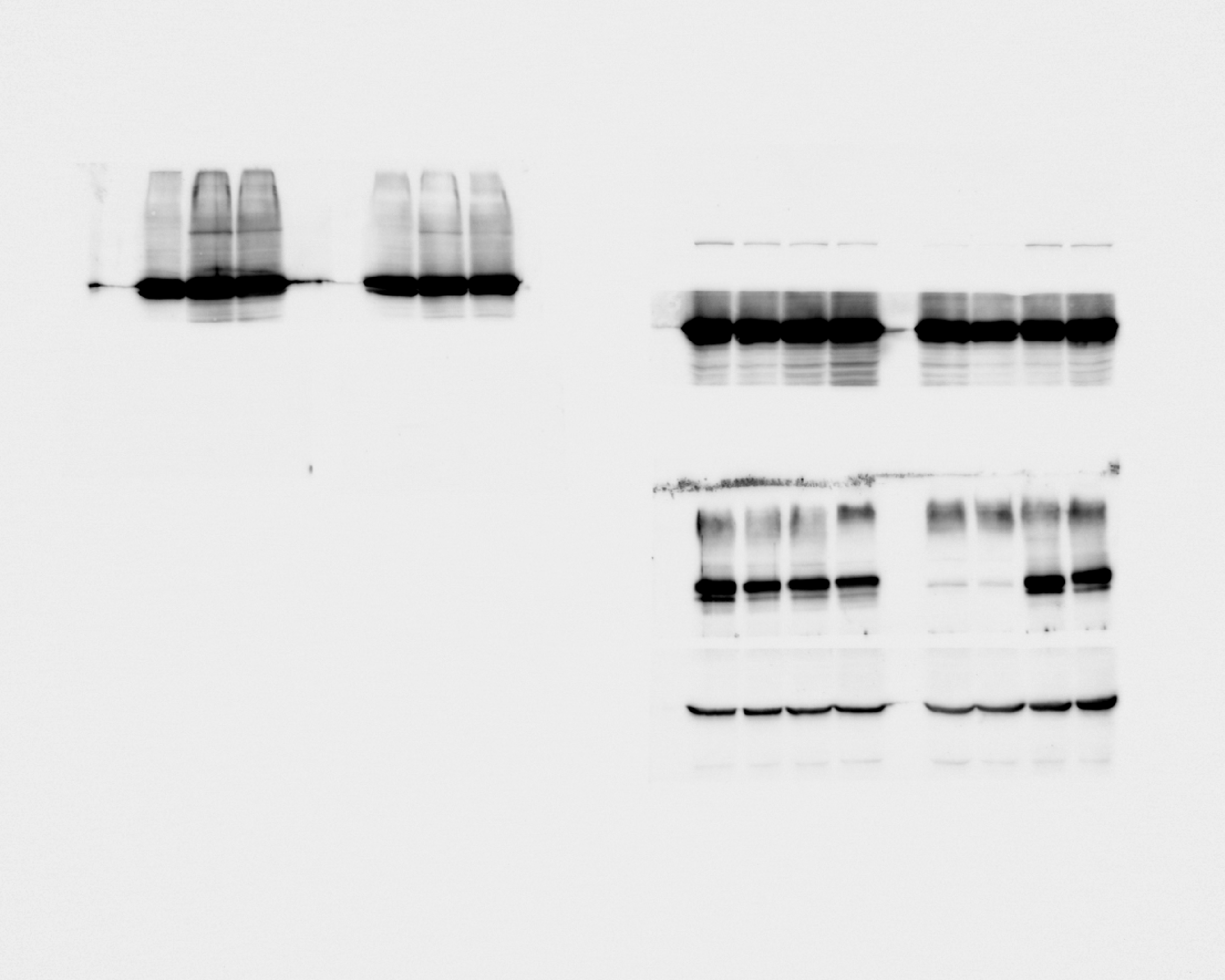

Supplement: Figure 7—figure supplement 1—source data 2. [file elife-74447-fig7-figsupp1-data2.zip › Figure 7-figure supplement 1-source data 2/Figure 7-figure supplement 1-source data 2.tif]

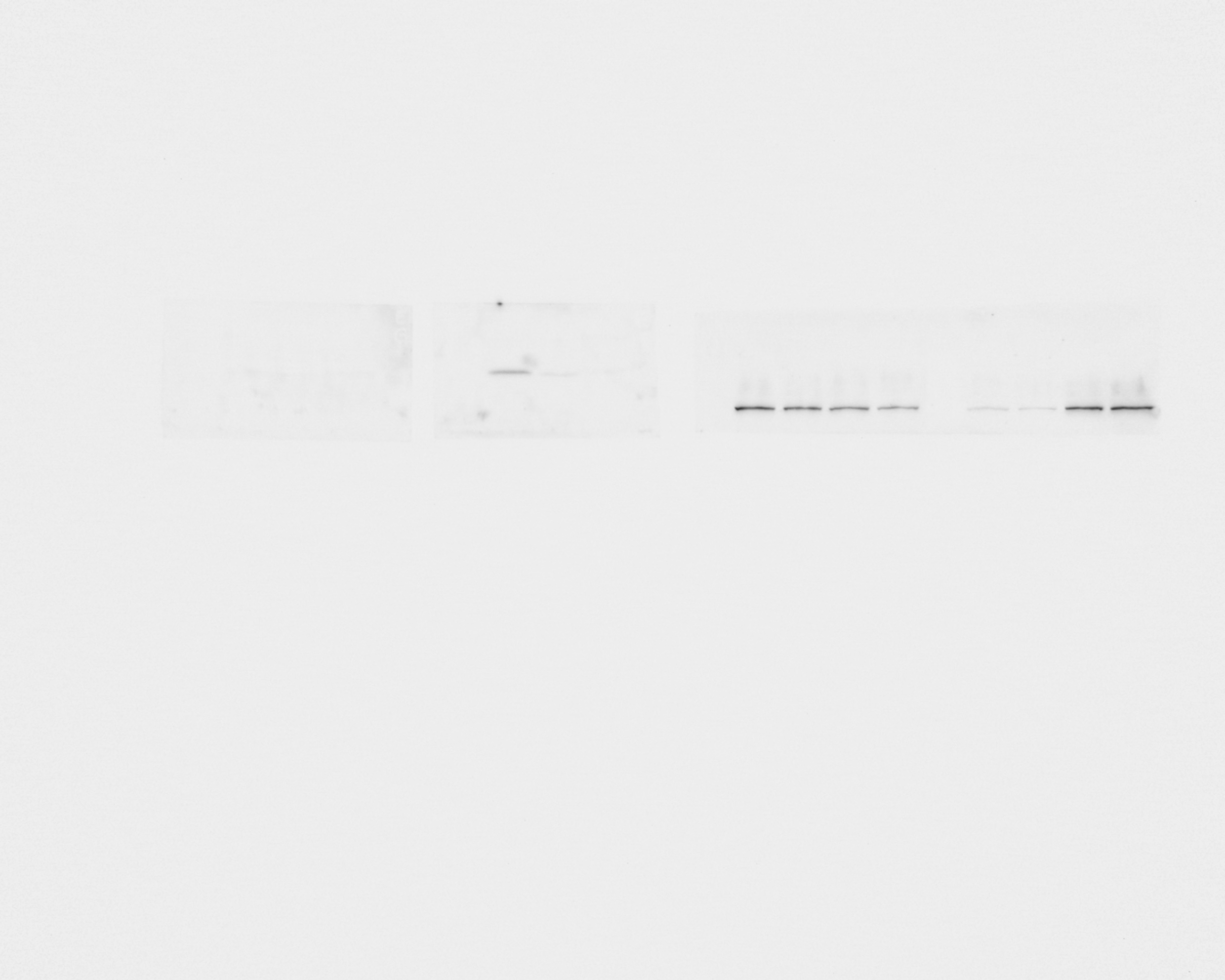

Supplement: Figure 7—figure supplement 1—source data 3. [file elife-74447-fig7-figsupp1-data3.zip › Figure 7-figure supplement 1-source data 3/Figure 7-figure supplement 1-source data 3.tif]

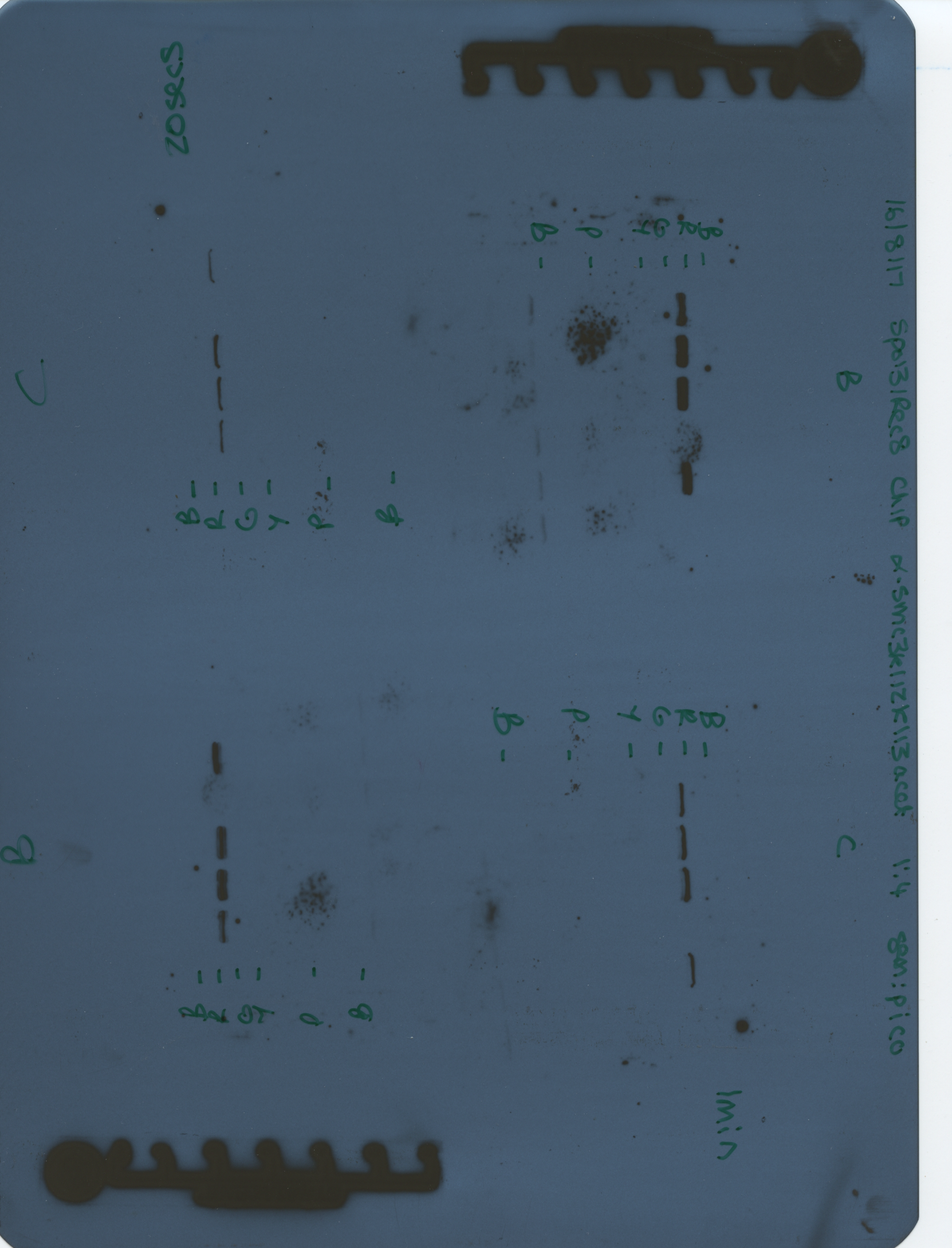

Supplement: Figure 8—figure supplement 2—source data 1. [file elife-74447-fig8-figsupp2-data1.zip › Figure 8-figure supplement 2-source data 1/Figure 8-figure supplement 2-source data 1.tiff]

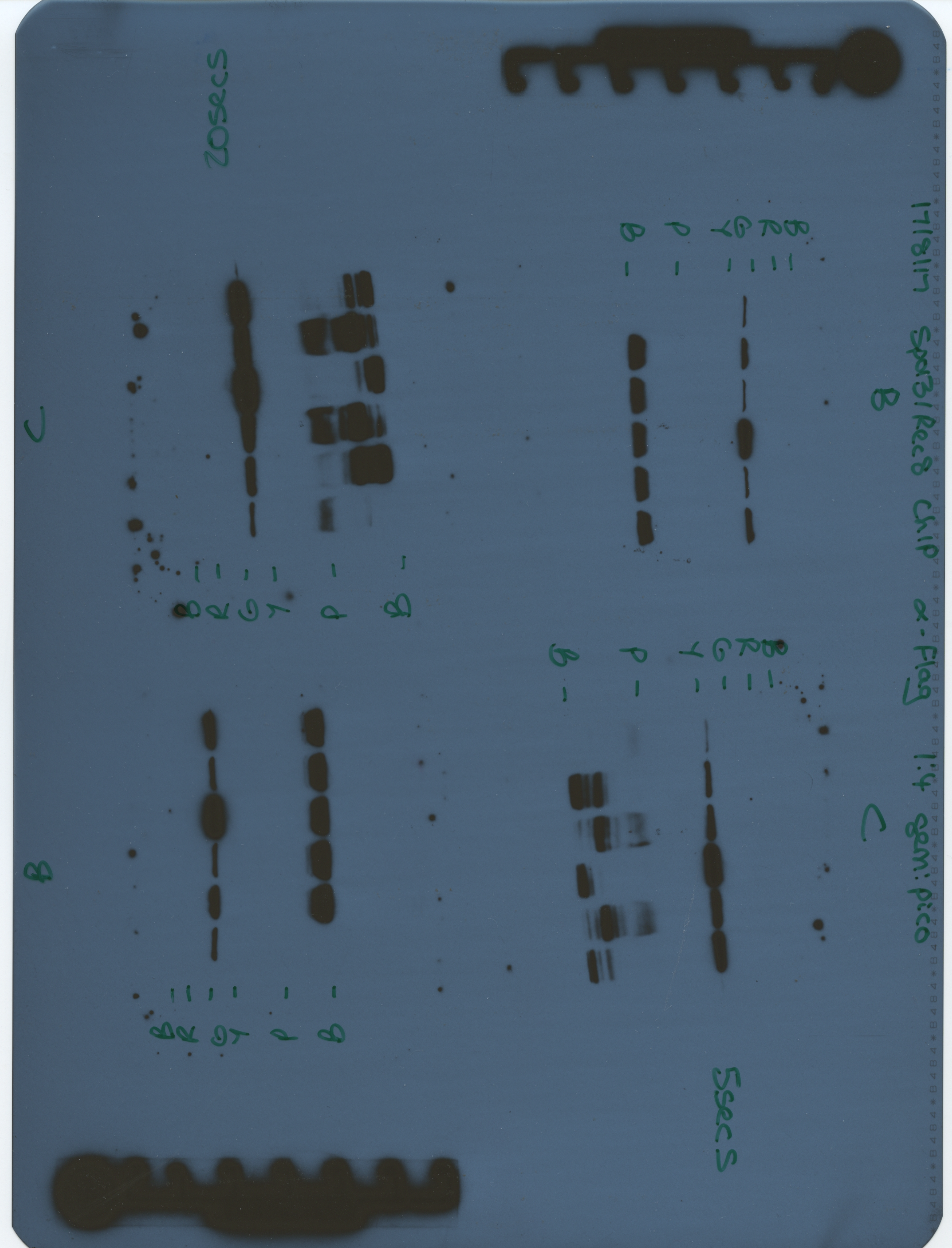

Supplement: Figure 8—figure supplement 2—source data 2. [file elife-74447-fig8-figsupp2-data2.zip › Figure 8-figure supplement 2-source data 2/Figure 8-figure supplement 2-source data 2.tiff]

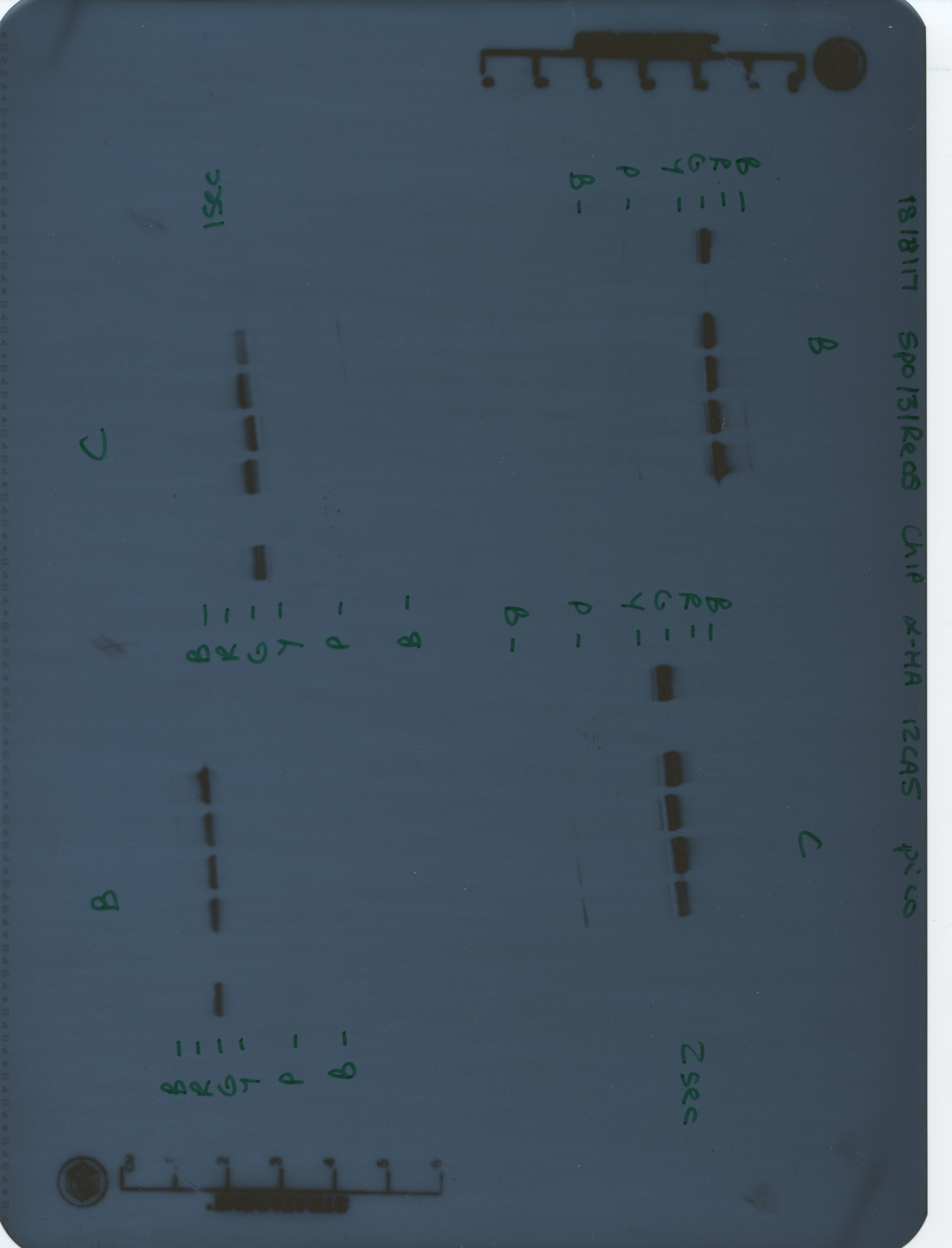

Supplement: Figure 8—figure supplement 2—source data 3. [file elife-74447-fig8-figsupp2-data3.zip › Figure 8-figure supplement 2-source data 3/Figure 8-figure supplement 2-source data 3.tiff]

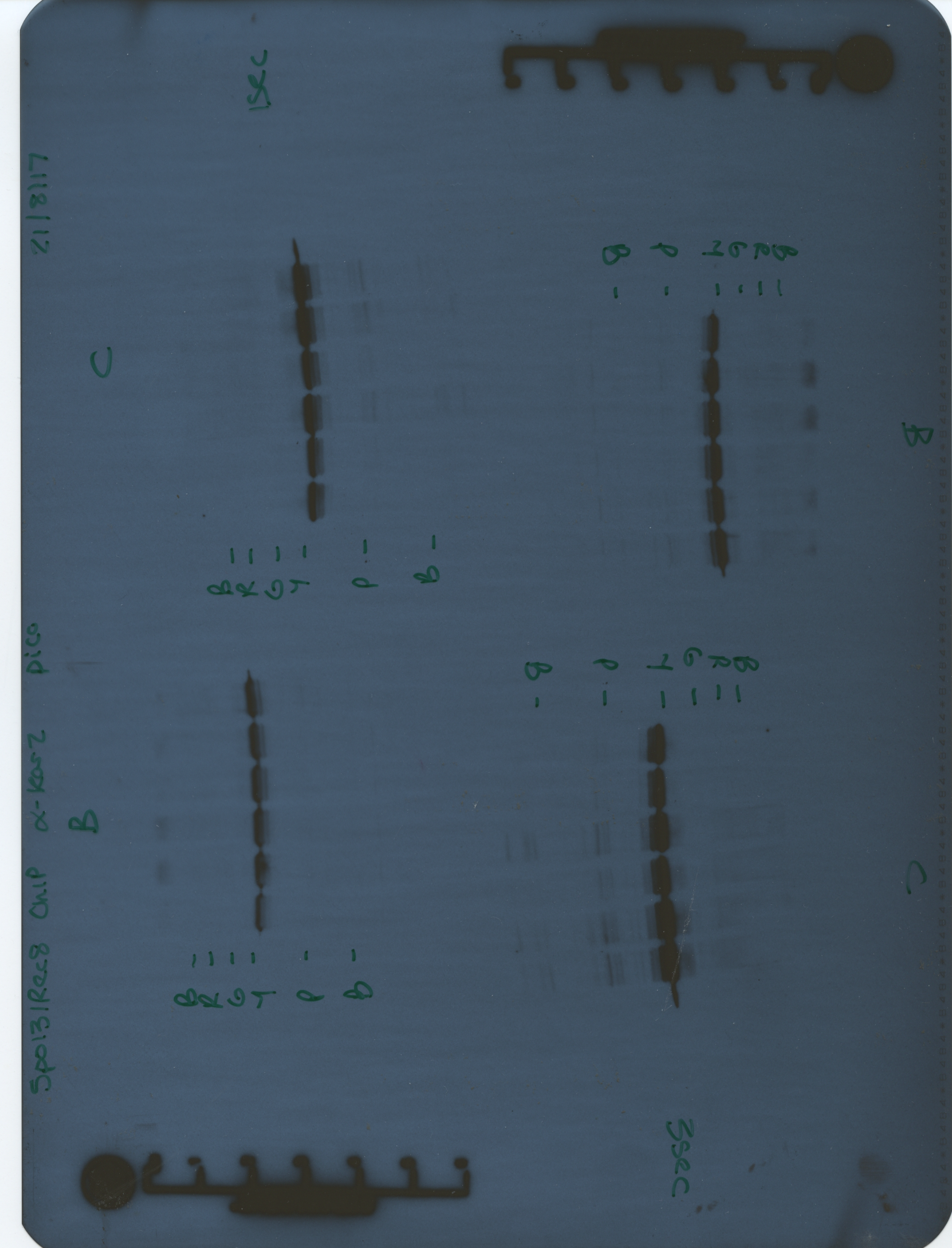

Supplement: Figure 8—figure supplement 2—source data 4. [file elife-74447-fig8-figsupp2-data4.zip › Figure 8-figure supplement 2-source data 4/Figure 8-figure supplement 2-source data 4.tiff]

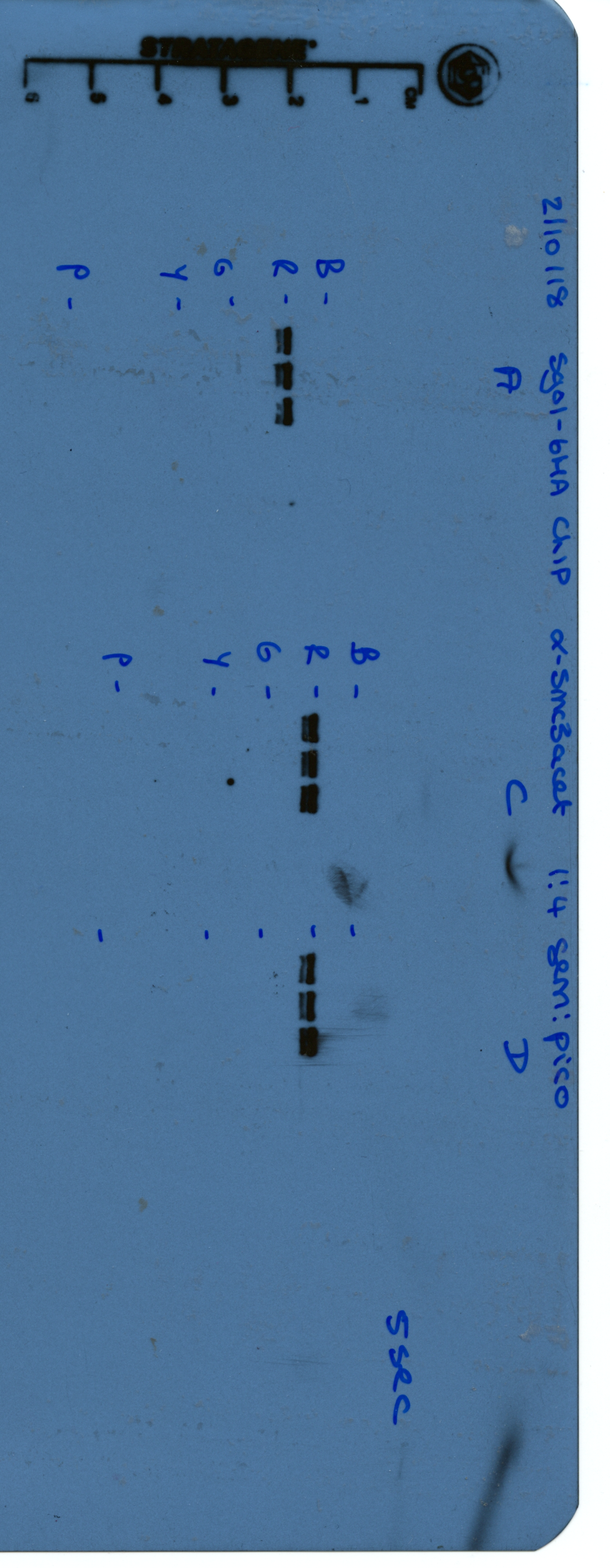

Supplement: Figure 8—figure supplement 2—source data 5. [file elife-74447-fig8-figsupp2-data5.zip › Figure 8-figure supplement 2-source data 5/Figure 8-figure supplement 2-source data 5.tif]

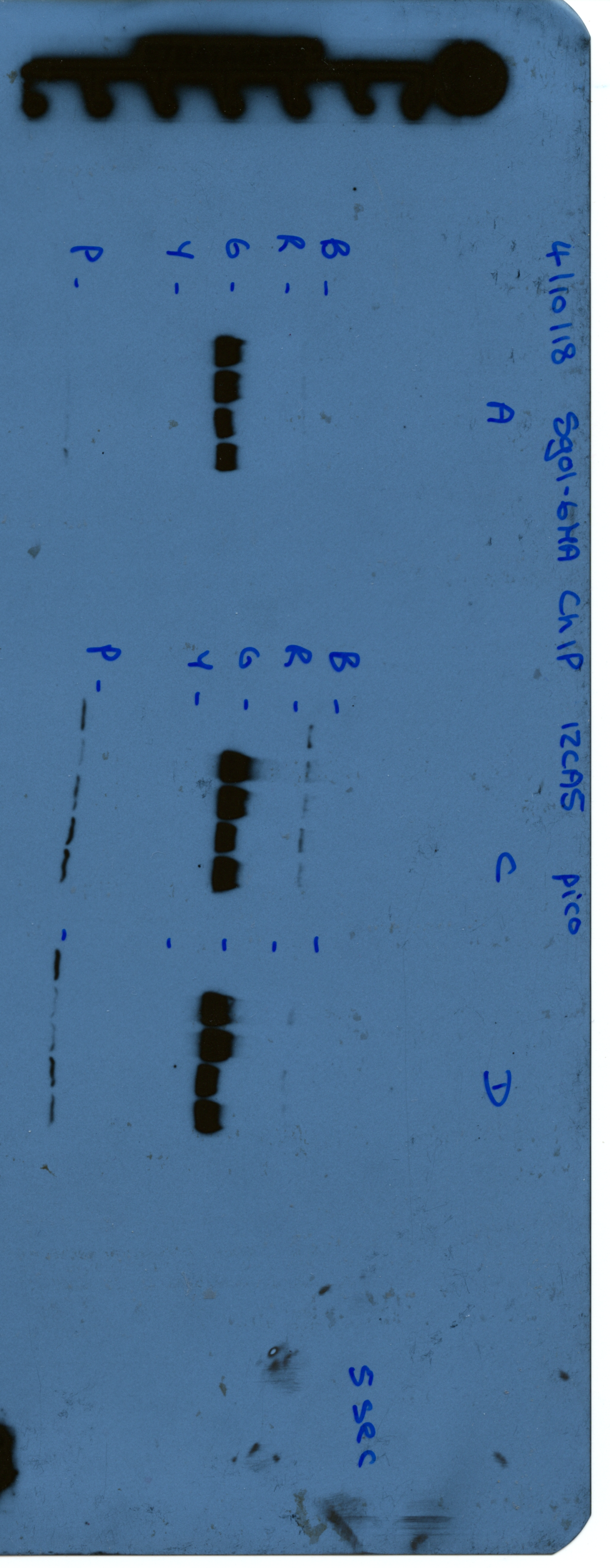

Supplement: Figure 8—figure supplement 2—source data 6. [file elife-74447-fig8-figsupp2-data6.zip › Figure 8-figure supplement 2-source data 6/Figure 8-figure supplement 2-source data 6.tif]

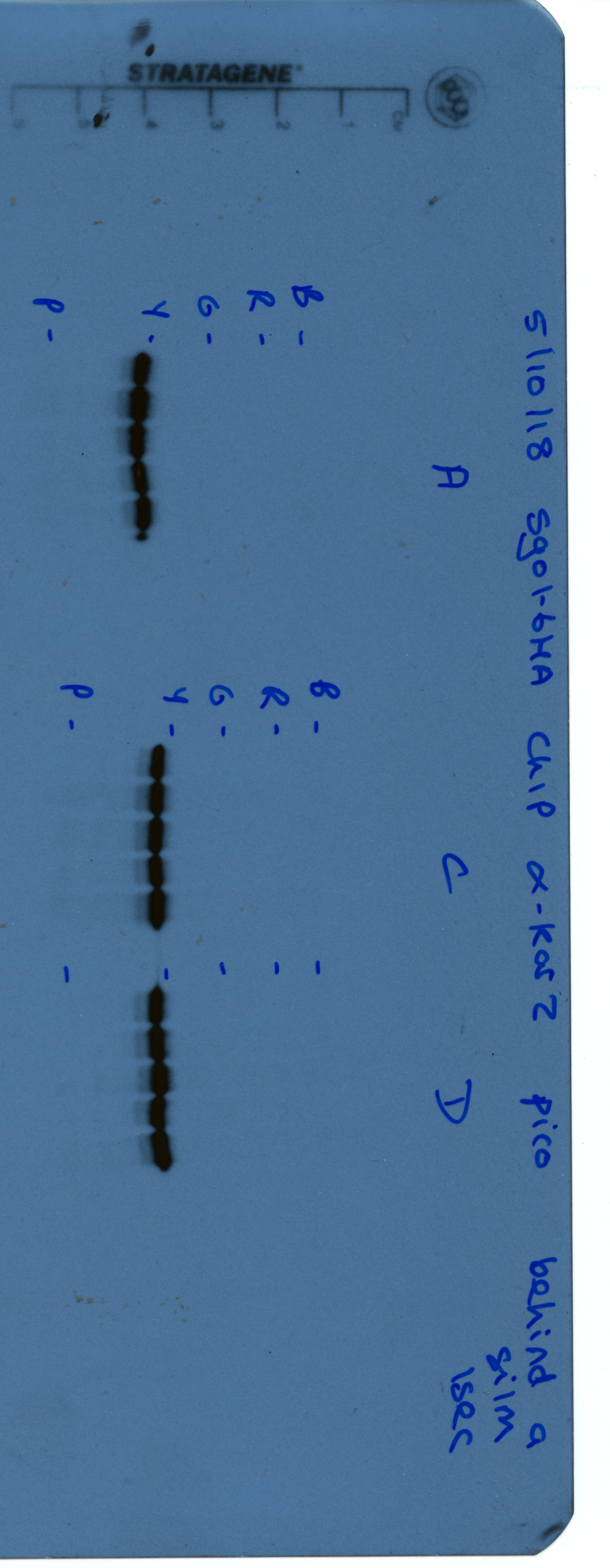

Supplement: Figure 8—figure supplement 2—source data 7. [file elife-74447-fig8-figsupp2-data7.zip › Figure 8-figure supplement 2-source data 7/Figure 8-figure supplement 2-source data 7.tif]

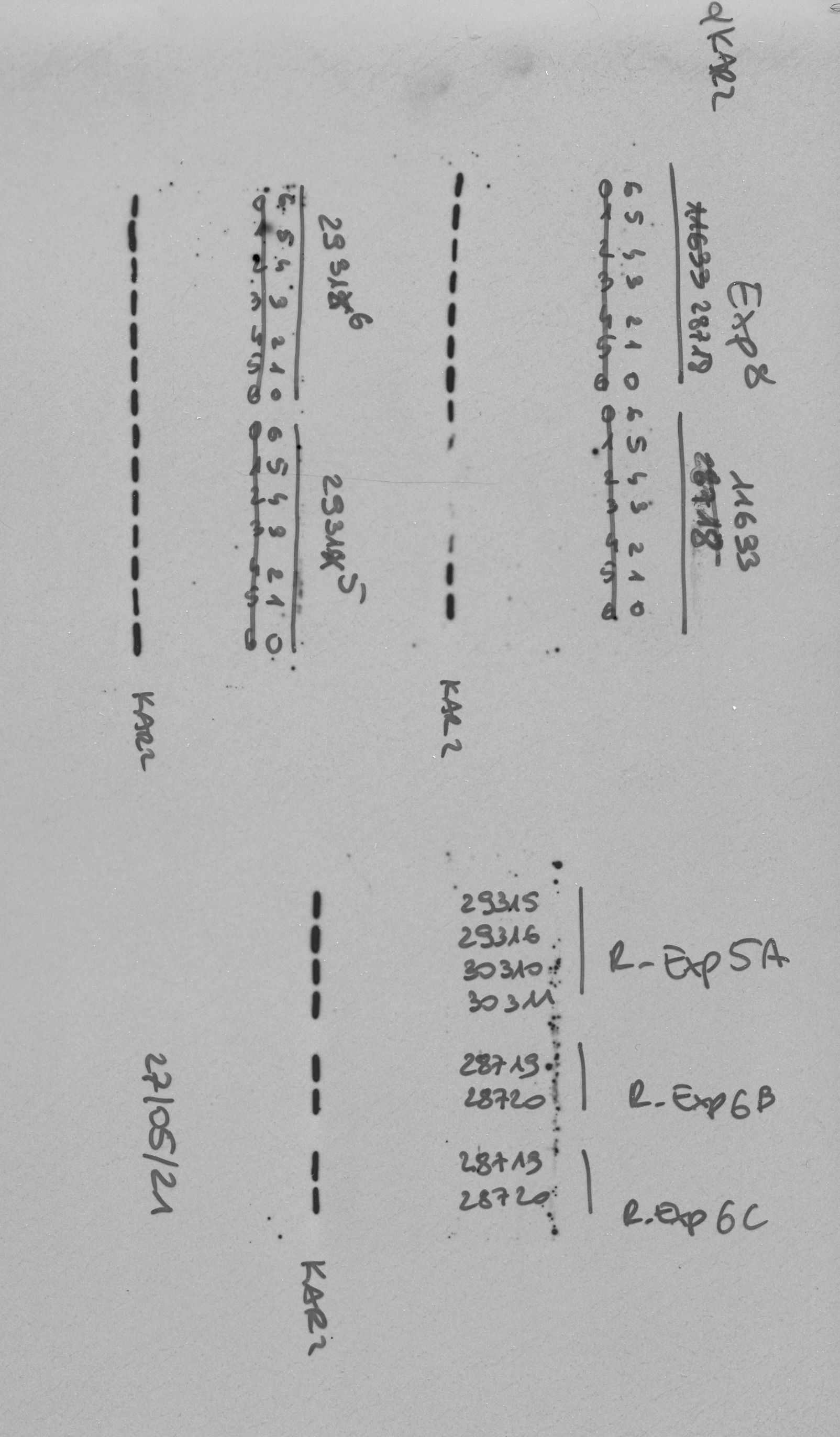

Supplement: Figure 9—source data 1. [file elife-74447-fig9-data1.zip › Figure 9-source data 1/Figure 9-source data 1.tif]

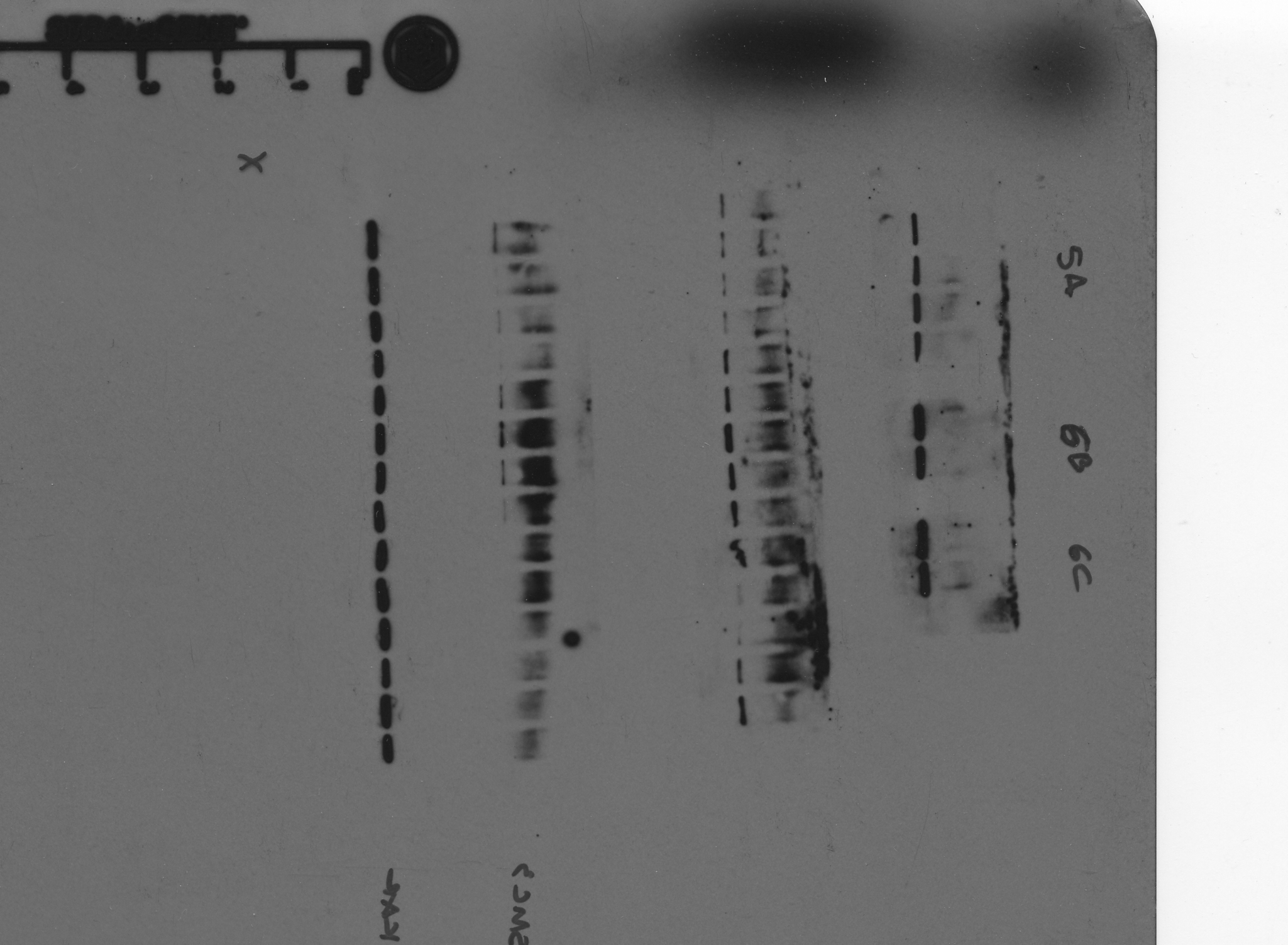

Supplement: Figure 9—source data 2. [file elife-74447-fig9-data2.zip › Figure 9-source data 2/Figure 9-source data 2.tif]

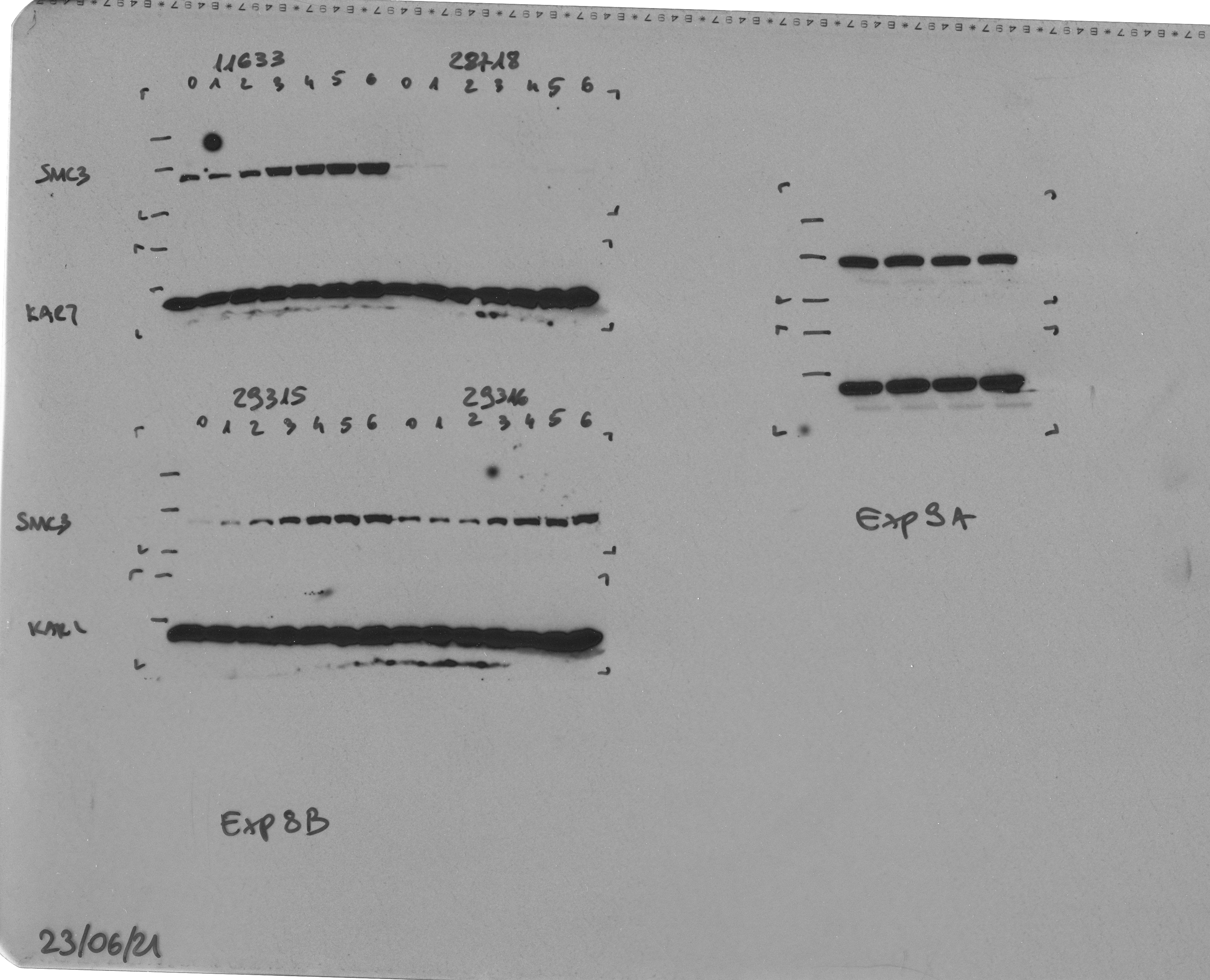

Supplement: Figure 9—figure supplement 1—source data 1. [file elife-74447-fig9-figsupp1-data1.zip › Figure 9-figure supplement 1-source data 1/Figure 9-figure supplement 1-source data 1.tif]

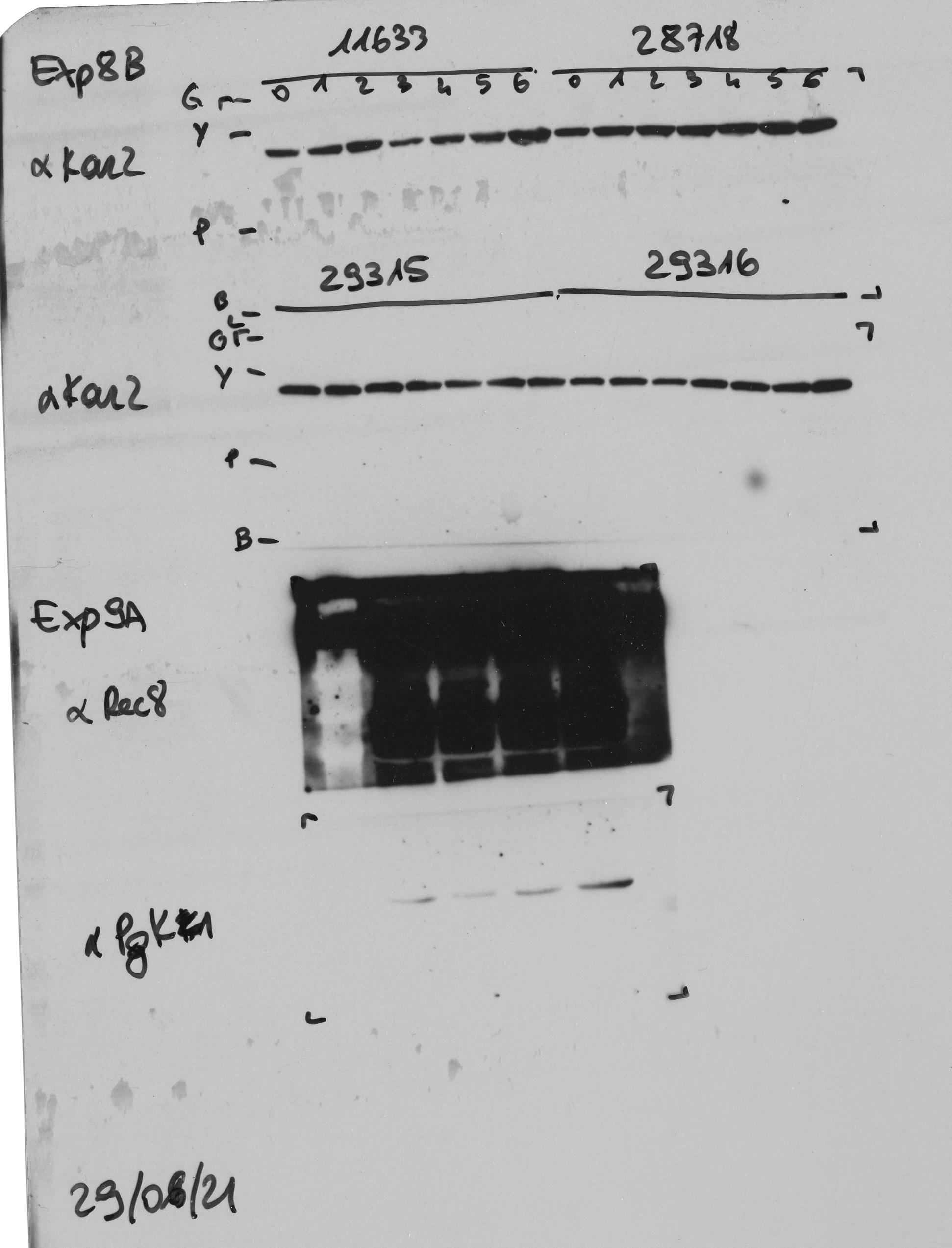

Supplement: Figure 9—figure supplement 1—source data 2. [file elife-74447-fig9-figsupp1-data2.zip › Figure 9-figure supplement 1-source data 2/Figure 9-figure supplement 1-source data 2.tif]

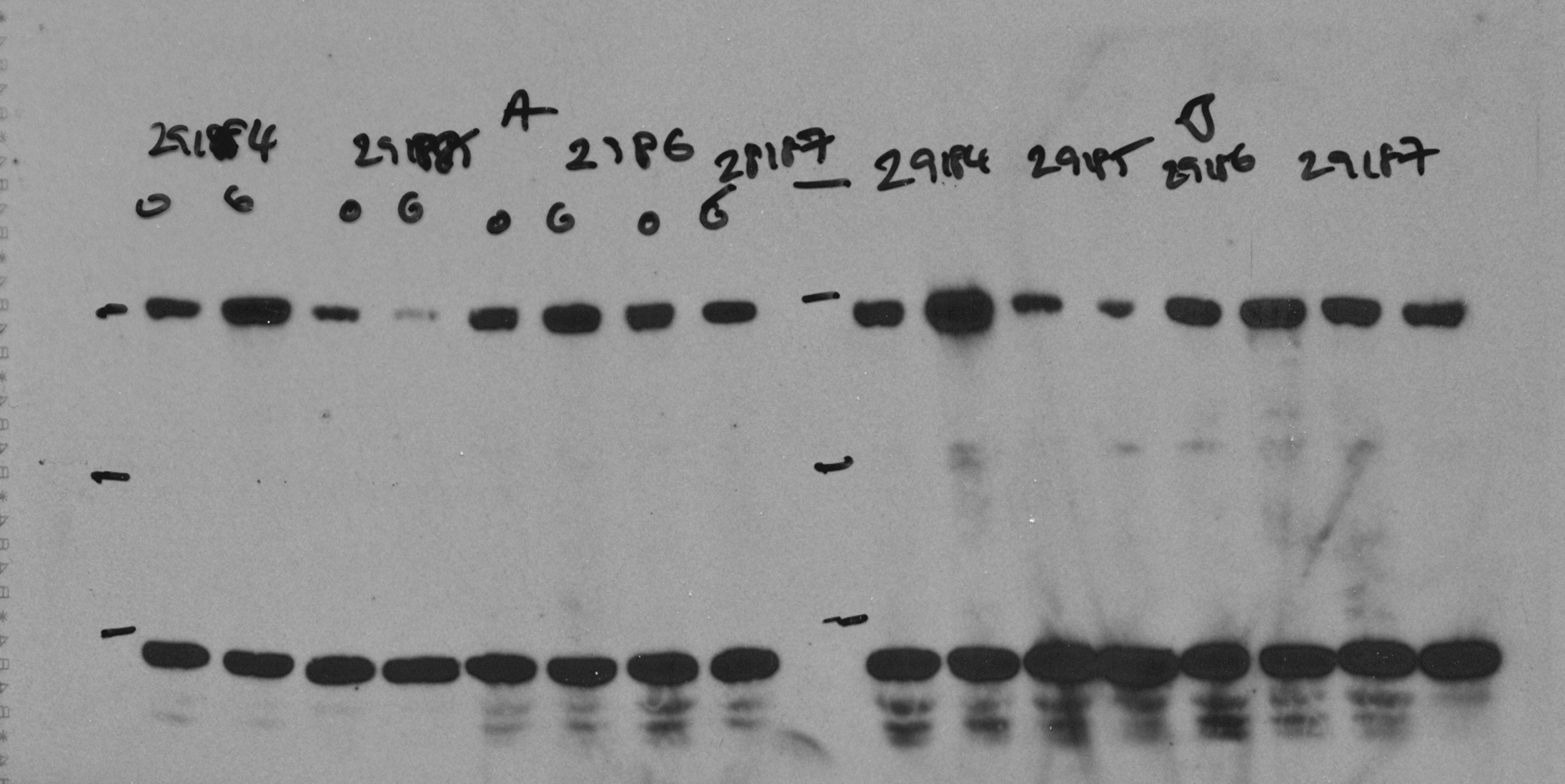

Supplement: Figure 9—figure supplement 2—source data 1. [file elife-74447-fig9-figsupp2-data1.zip › Figure 9-figure supplement 2-source data 1/Figure 9-figure supplement 2-source data 1.tif]

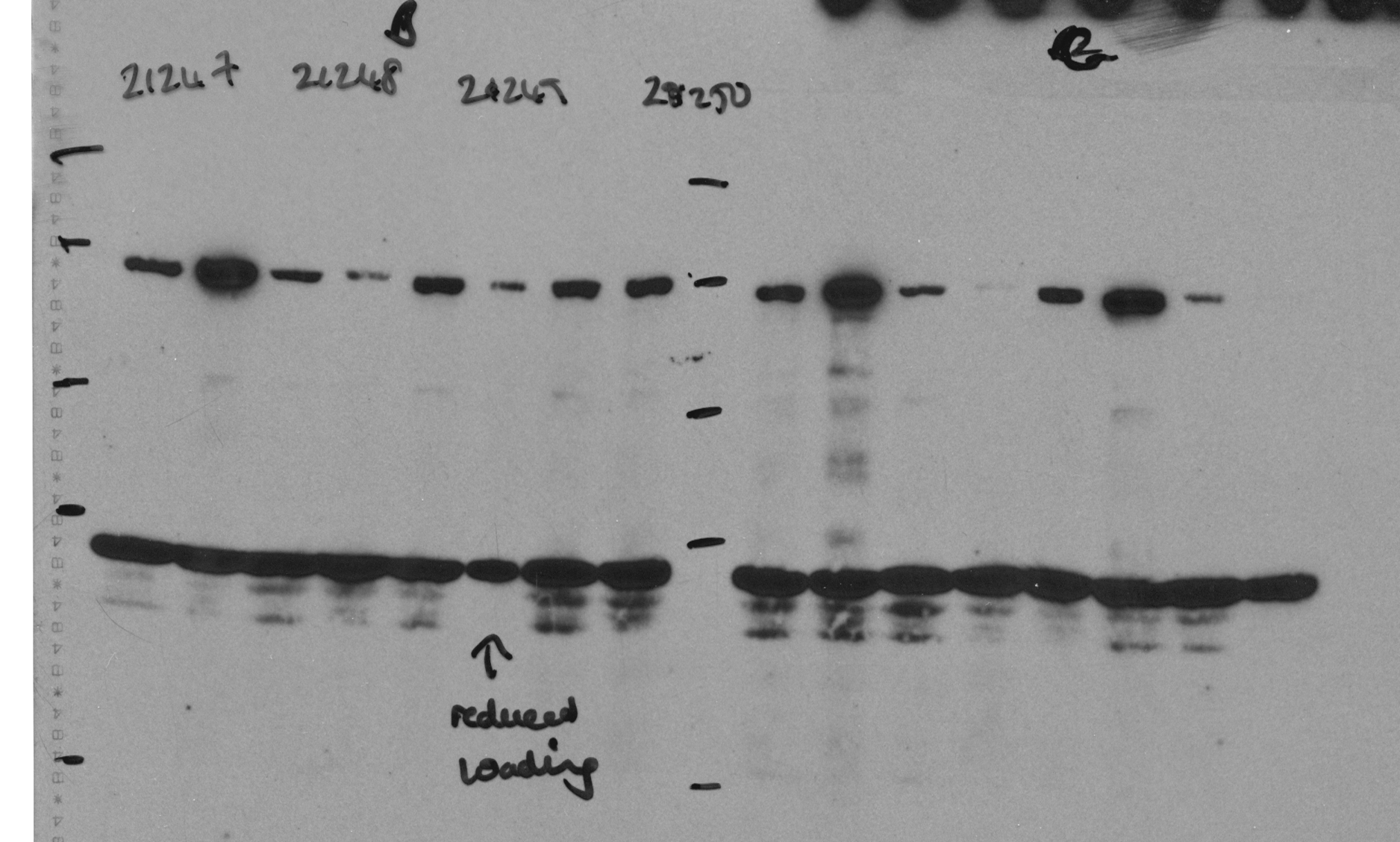

Supplement: Figure 9—figure supplement 2—source data 2. [file elife-74447-fig9-figsupp2-data2.zip › Figure 9-figure supplement 2-source data 2/Figure 9-figure supplement 2-source data 2.tif]
