# Supplementary material for: Eco1-dependent cohesin acetylation anchors chromatin loops and cohesion to define functional meiotic chromosome domains
Source: eLife. 2022 Feb 1;11:e74447. doi: 10.7554/eLife.74447 (PMC8856730; doi:10.7554/eLife.74447)
Supplement: Transparent reporting form [file elife-74447-transrepform1.pdf]

Westbrook Centre
Milton Road
Cambridge CB4 1YG, UK

P 01223 855340
W elifesciences.org
T @elife

eLife's transparent reporting form

We encourage authors to provide detailed information *within their submission* to facilitate the interpretation and replication of experiments. Authors can upload supporting documentation to indicate the use of appropriate reporting guidelines for health-related research (see EQUATOR Network), life science research (see the BioSharing Information Resource), or the ARRIVE guidelines for reporting work involving animal research. Where applicable, authors should refer to any relevant reporting standards documents in this form.

If you have any questions, please consult our Journal Policies and/or contact us: editorial@elifesciences.org.

Sample-size estimation

- You should state whether an appropriate sample size was computed when the study was being designed
- You should state the statistical method of sample size computation and any required assumptions
- If no explicit power analysis was used, you should describe how you decided what sample (replicate) size (number) to use

Please outline where this information can be found within the submission (e.g., sections or figure legends), or explain why this information doesn't apply to your submission:

Sample sizes used are typical of similar studies and were selected to ensure robust and statistically significant comparisons.
No statistical method for sample size computation was used. For Hi-C and ChIP experiments, cultures containing several million cells were used to determine the genome average. For western blotting timecourses several million cells were harvested at each timepoint. For microscopy experiments on fixed cells, at least 100 cells were scored for each timepoint in each biological repeat. For live cell imaging at least 100 cells were analysed for each biological repeat. For spore viability assay, 50 tetrads were scored for each condition. For flow cytometry analysis, at least 20,000 events were acquired. Sample sizes are provided in the figures or figure legends.

Replicates

- You should report how often each experiment was performed
- You should include a definition of biological versus technical replication
- The data obtained should be provided and sufficient information should be provided to indicate the number of independent biological and/or technical replicates
- If you encountered any outliers, you should describe how these were handled
- Criteria for exclusion/inclusion of data should be clearly stated
- High-throughput sequence data should be uploaded before submission, with a private link for reviewers provided (these are available from both GEO and ArrayExpress)

Please outline where this information can be found within the submission (e.g., sections or figure legends), or explain why this information doesn't apply to your submission:

At least three biological replicates (on different biological samples) of each fixed-cell imaging timecourse were performed and the average is shown. At least two biological replicates of each western blotting timecourse were performed, with the exception of experiment in Figure 1E and Figure 1–figure supplement 2 that was performed once, in two different backgrounds (wild type and *spo11Δ*). A representative replicate is shown. Sporulation assays were performed once. For live imaging experiments, at least two biological replicates were performed and one is shown. For ChIP-qPCR experiments at least three biological replicates were performed, and for each biological replicate qPCR reactions were carried out in technical triplicate (repeated measurements of the same sample). Hi-C and most ChIP-Seq experiments are a single replicate for each condition, however, all chromosomal arms and pericentromeres show the same pattern. Replicates were excluded only if adequate arrest or synchronicity conditions were not met. Number of replicates are provided in the figure legends and in the Materials and methods section. High-throughput sequence data have been deposited on GEO under accession number GSE185021 and are available for reviewers with the following secure token: wzireciibrsvxet.

Statistical reporting

- Statistical analysis methods should be described and justified
- Raw data should be presented in figures whenever informative to do so (typically when N per group is less than 10)
- For each experiment, you should identify the statistical tests used, exact values of N, definitions of center, methods of multiple test correction, and dispersion and precision measures (e.g., mean, median, SD, SEM, confidence intervals; and, for the major substantive results, a measure of effect size (e.g., Pearson's r, Cohen's d)
- Report exact p-values wherever possible alongside the summary statistics and 95% confidence intervals. These should be reported for all key questions and not only when the p-value is less than 0.05.

Please outline where this information can be found within the submission (e.g., sections or figure legends), or explain why this information doesn't apply to your submission:

For fixed-cell imaging timecourses and for ChIP-qPCR experiments, mean and standard error are shown; significance in comparison to the wild type control was estimated with paired student T test where indicated. For Hi-C and ChIP-seq pile-up plots, average, standard error and 95% confidence interval of all 16 centromeres or 32 pericentromeres or 32 arm peak positions are shown. For all other experiments, a representative replicate is shown and no statistical analysis was performed. Statistical methods are provided in the figure legends where applicable.

(For large datasets, or papers with a very large number of statistical tests, you may upload a single table file with tests, Ns, etc., with reference to sections in the manuscript.)

Group allocation

- Indicate how samples were allocated into experimental groups (in the case of clinical studies, please specify allocation to treatment method); if randomization was used, please also state if restricted randomization was applied
- Indicate if masking was used during group allocation, data collection and/or data analysis

Please outline where this information can be found within the submission (e.g., sections or figure legends), or explain why this information doesn't apply to your submission:

Group allocation is not applicable to genetic comparisons. Yeast strains used are isogenic clones, with only the indicated mutations compared to wild type. Strains genotypes are provided in Supplementary table 1. All cells analysed in population and single-cell analyses were derived from the same verified clone. In experiments where drug treated (rapamycin) and untreated (DMSO) cells were compared, cultures were grown from the same yeast isolate cultured in the same flask and split prior to rapamycin or DMSO addition. In all experiments flasks and samples were labelled with strain numbers and no other identifying information. For microscopy scoring, samples were labelled with strain numbers and no other identifying information. Acquisition and analysis of live-imaging data were performed by different authors; analysis was performed on data classified by strain number and no other identifying information. Analysis of Hi-C and ChIP-seq data were performed blindly, with no identifying information on the datasets.

Additional data files ("source data")

- We encourage you to upload relevant additional data files, such as numerical data that are represented as a graph in a figure, or as a summary table
- Where provided, these should be in the most useful format, and they can be uploaded as "Source data" files linked to a main figure or table
- Include model definition files including the full list of parameters used
- Include code used for data analysis (e.g., R, MatLab)
- Avoid stating that data files are "available upon request"

Please indicate the figures or tables for which source data files have been provided:

Codes used for ChIP-seq and Hi-C data analysis are available at

https://github.com/danrobertson87/Barton_2021

eLife Sciences Publications, Ltd is a limited liability non-profit non-stock corporation incorporated in the State of Delaware, USA, with company number 5030732, and is registered in the UK with company number FC030576 and branch number BR015634 at the address Westbrook Centre, Milton Road, Cambridge CB4 1YG.
